# Effect of early postnatal supplementation of newborns with probiotic strain *E. coli* O83:K24:H31 on allergy incidence, dendritic cells, and microbiota

**DOI:** 10.3389/fimmu.2022.1038328

**Published:** 2023-01-09

**Authors:** Lenka Súkeníková, Viktor Černý, Tomáš Thon, Radka Roubalová, Zuzana Jirásková Zákostelská, Olga Novotná, Petra Petrásková, Kristýna Boráková, Ingrid Kocourková, Rája Lodinová-Žádníková, Zdeněk Musil, Libuše Kolářová, Ludmila Prokešová, Zdeněk Valenta, Jiří Hrdý

**Affiliations:** ^1^ Institute of Immunology and Microbiology, First Faculty of Medicine, Charles University, Prague, Czechia; ^2^ Institute of Microbiology, Academy of Sciences, Prague, Czechia; ^3^ Department of Neonatology, Institute for the Care of Mother and Child, Prague, Czechia; ^4^ Institute of Biology and Medical Genetics, First Faculty of Medicine, Charles University, Prague, Czechia; ^5^ Department of Statistical Modelling, Institute of Computer Science of the Czech Academy of Sciences, Prague, Czechia

**Keywords:** probiotic, dendritic cell, IL-10, CD83, *Escherichia coli* O83:K24:H31, flow cytometry, allergy, cord blood

## Abstract

**Introduction:**

Probiotic administration seems to be a rational approach to promote maturation of the neonatal immune system. Mutual interaction of the microbiota with the host immune system is critical for the setting of appropriate immune responses including a tolerogenic one and thevmaintenance of homeostasis. On the other hand, our knowledge on the modes of actions of probiotics is still scarce.

**Methods:**

In our study, probiotic strain Escherichia coli O83:K24:H31 (EcO83) was administered to neonates of allergic mothers (AMs; neonates with increased risk for allergy development) within 48 h after the delivery, and the impact of this early postnatal supplementation on allergy incidence and selected immune markers has been analyzed 10 years after the primary EcO83 administration.

**Results:**

We have observed decreased allergy incidence in 10-year-old children supplemented with EcO83 (13 of 52 children were allergic) in comparison with non-supplemented children of AMs (16 of 42 children were allergic). The early postnatal EcO83 supplementation appeared to limit the allergy in the high-risk group (children of AMs) compared to that in the low-risk group (children of healthy mothers). Dendritic cells (DCs) in the peripheral blood of EcO83-supplemented children do not differ significantly in cell surface presence of CD83. The immunomodulatory capacity of EcO83 on DCs was tested in vitro as well. Both directly isolated myeloid and in vitro monocyte-derived DCs from cord blood increased CD83 expression together with interleukin (IL)-10 secretion after EcO83 stimulation. The effect of early postnatal EcO83 supplementation on the microbiota composition of 10-year-old children was characterized by next-generation sequencing, and we have not observed significant changes in the microbiota composition of EcO83-supplemented and non-supplemented children at the age of 10 years.

**Conclusions:**

Early postnatal EcO83 supplementation appears to lower allergy incidence in children of AMs. It seems that the beneficial effect of EcO83 is mediated via modulation of DC functional capacities without impacting the microbiota composition. Larger-scale studies will be necessary to confirm these preliminary findings.

## 1 Introduction

A dramatic increment of the incidence of allergic diseases has been reported, raising a need to identify early predictive markers pointing to an increased risk of allergy development so that suitable preventive measures can be introduced. Various parameters characterizing immune responses have been proposed, including immunoglobulin E (IgE) levels in cord blood (1), T helper cell type 1 (Th1) and Th2 cytokine concentrations in cord blood sera and gene expression in cord blood cells (2), cytokine secretion and proliferation of cord blood cells in response to stimulation with allergens or polyclonal antigens (3–7), concentration of immunoregulatory cytokines (8), proportion of regulatory T cells (Treg) (9–11), and changes in epigenetic regulation (12, 13). Some studies focused on immunological characteristics of maternal milk of healthy and allergic women (14–16). Despite the effort of different research groups to find some early prognostic markers or a group of markers, it seems that the maternal allergy status remains the marker with the best predictive value of future allergy development (17).

With the growing knowledge on the impact of the microbiota on host immune responses, the key role of pioneering microorganisms colonizing the neonatal gut in the maturation of the newborn’s immune system has been acknowledged (18–20). Since it was documented that children delivered vaginally or by Cesarean section have different microbiota (21), the mode of delivery could contribute to the appropriate development of immune responses in the key neonatal period. The presence of distinct microbiota colonizing the relatively sterile neonatal gut strongly affects the maturation of the newborn immune system including the setting of tolerogenic immune responses to relatively innocuous environmental antigens and compounds of the microbiota (22).

Dendritic cells (DCs), as the professional antigen-presenting cells, play a key role in the priming and polarization of immune responses. In the context of allergic diseases mediated by IgE, after antigen engulfment, processing and presentation by DCs are preferably promoting Th2 immune responses with typical Th2 cytokine production [interleukin (IL)-4, IL-13], leading to the secretion of IgE from B cells in predisposed individuals. In humans, several different subsets of DC were identified. The most prominent are myeloid DCs (mDCs) characterized by CD11c expression. The other major group of DCs is represented by plasmacytoid DCs (pDCs), sometimes called interferon-producing cells (IPCs), with the typical marker CD123 (23). Interstitial, follicular, and tolerogenic DCs represent important functional subtypes with distinct tissue localization and biological significance. In the skin, Langerhans cells are present as the most abundant DC type. Both mDCs and pDCs were shown to be increased in human lungs after allergen challenge (24). In addition, the capacity of both mDCs and pDCs to stimulate the allergen-specific Th2 immune response was demonstrated (25). On the contrary, only mDCs are capable of promoting the innate lymphoid cell type 2 (ILC2) contributing to allergy development while activated pDCs rather inhibit ILC2 *via* IL-6 secretion (26). The key role of mDCs in allergic disease development has been demonstrated in an experimental mouse model in which only mDCs were able to induce asthma in a dose-dependent manner, while pDC administration had only a minor effect on asthma development (27). In another report, compartment imbalance with pDC predominance was observed in patients suffering from atopic dermatitis (28). Similarly, the key role of pDCs in setting the tolerance to allergens has been highlighted, and its impairment was associated with asthma development (29). ST2 (receptor for key Th2 cytokine IL-33) was upregulated on both mDCs and pDCs of patients suffering from allergic rhinitis (30). Thus, the role of mDCs in allergy development is well documented, while reports on the role of pDCs in allergy development and propagation are contradictory. In addition, it seems that the role of particular DC subsets is tissue dependent, and therefore, further study is needed to understand the role of both main DC subsets in allergy.

The functional capacity of DCs can be modified by bacteria (e.g., probiotics) and/or bacterial products, including bacterial lysates (31–33). Bacteria-primed DCs polarize immune responses differently depending on the bacteria used for DC priming (32). Some bacteria are potent inducers of Th1 (34), Th2 (35), Th17 (36, 37), or Treg (38). Bacteria with specific immunomodulatory capacity can be exploited in the prevention or treatment of various diseases using different experimental approaches. In the context of allergic diseases, it is highly desirable to use bacterial strains with the capacity to limit Th2 and promote Th1 together with Treg because newborns have generally immature immune systems with predominant Th2 immune responses. Some promising probiotics have already been identified (e.g., 39, 40). On the other hand, excessive Th1 immune responses could lead to the development of other disorders such as autoimmune diseases or Crohn’s disease.

In our previous study, decreased allergy incidence was observed 10 and 20 years after early postnatal administration of *Escherichia coli* O83:K24:H31 (EcO83) (41). These intriguing observations led us to start a new study where selected immune parameters were prospectively followed. In the current report, we are presenting the impact of early postnatal supplementation of newborns with EcO83 on allergy incidence at the age of 10 years. To address how this early postnatal EcO83 administration could influence the immune system, we determined the dynamics of cytokine concentrations in peripheral blood up to 10 years. In addition, the maturational status of DCs and selected functional characteristics of Treg were analyzed at the age of 10 years. To better understand possible modes of actions of EcO83 on the neonatal immune system, cord blood DCs were stimulated with EcO83 *in vitro*. The possible impact of early postnatal EcO83 supplementation on the microbiota development has been analyzed as well. It seems that early postnatal administration of EcO83 may prevent allergy development and impacts the functional capacity of both Treg and DCs. We were able to demonstrate the capacity of EcO83 to induce DC maturation together with the promotion of immunoregulatory cytokine secretion without impacting the microbiota composition at the age of 10 years.

## 2 Materials and methods

### 2.1 Subjects

Pregnant women without complications during pregnancy were included in the study after providing a signed written informed consent. Based on allergic status, the women were divided into two groups. Pregnant women with clinical manifestations of allergy persisting for a period longer than 24 months before pregnancy were considered allergic mothers (AMs). We acknowledge that the allergic group is quite heterogeneous, consisting of women suffering from various kinds of food and pollen allergy, allergy to insects, eczema, and/or asthma. Exclusion criteria were the presence of inflammatory bowel disease, any kind of autoimmune disease, celiac disease, cancer, transplantation, blood transfusions, repeated abortions, children conceived after *in vitro* fertilization, or multiparous pregnancy. Antibiotic administration during the delivery was not among the exclusion criteria [one mother received antibiotics in the group of non-supplemented children of healthy mothers (N HM), three mothers received antibiotics in the group of non-supplemented children of allergic mothers (N AM), and one mother received antibiotics in the group of probiotic-supplemented children of allergic mothers (S AM)]. The mothers were recruited from Prague or its suburban area, and all children were delivered vaginally. The study was approved by the Ethical Committee of the Institute for the Care of Mother and Child (Prague, Czechia).

### 2.2 Evaluation of the effect of early postnatal supplementation with *Escherichia coli* O83:K24:H31 on allergy incidence in 10-year-old children

The allergy status of children was confirmed by an allergist, with children who had specific IgE against allergen and/or positive skin prick test considered allergic. The full list of individual allergies is provided in **Supplementary Table S1**. Originally, 56 neonates of AMs (children at higher risk for allergy development) were supplemented with Colinfant Newborn containing probiotic strain EcO83 within 48 h after delivery as previously described (41–44) (S AM). In the group of non-supplemented children of allergic mothers (children at high risk of allergy development without probiotic treatment), 57 children were enrolled (N AM). The third group of N HM (children with relatively low risk for allergy development) consisted of 45 children. The children were followed prospectively, and here, we are reporting the results at the age of 10 years, i.e., 10 years after primary EcO83 administration. At this time point, approximately 5 ml of peripheral blood was collected from children into heparinized tubes during the control examination. At the age of 10 years, 23 children from the N HM group, 38 children from the N AM group, and 45 children from the S AM group took part in the regular checkup including blood collection. These three basic groups were divided based on the allergic status of the children at the age of 10 years into six subgroups, as indicated in **Supplementary Table S2**. The full list of maternal allergies (including allergen if known) is indicated in **Supplementary Table S3**. The characteristics of the neonates among the groups were comparable, as shown in **Supplementary Table S4**. We were furthermore able to obtain information about the allergic status of additional children only by telephonic contact, i.e., without biological material collection. This led to a minor increase of the number of children in every group (up to 32 children in N HM, 42 children in N AM, and 52 children in S AM). The final numbers of allergic children determined based on parental report and/or examination by allergist are shown in **Supplementary Table S5**.

### 2.3 The impact of early postnatal Escherichia coli O83:K24:H31 supplementation on cytokine and antibody production

#### 2.3.1 Cytokine determination in peripheral blood and cell culture supernatants

The concentration of cytokines in peripheral blood and cell culture supernatants was analyzed as previously described (43, 44). Briefly, cytokines in the plasma of peripheral blood of children and cytokines released by non-stimulated and stimulated DCs after 24 h of stimulation with EcO83 or lipopolysaccharide (LPS) (1 µg/ml, cat. no. L2654-1MG; Sigma-Aldrich, St. Louis, MO, USA) were detected by ELISA. Reagents for IL-4, IL-5, IL-6, IL-10, IL-13, interferon (IFN)-γ, and transforming growth factor (TGF)-β detection were purchased from R&D Systems (Minneapolis, MN, USA) (IL-4: primary antibody MAB 604, biotinylated secondary antibody BAF 204, recombinant standard protein 204-IL; IL-5: MAB 405, BAM 6051, 205-IL; IL-6: MAB 206, BAF 206, 206-IL; IL10: MAB 2172, BAF 2017, 217-IL; IL-13: MAB 213, BAF 285, 285-IL; IFN-γ: MAB 2852, BAF 285, 285-IF; TGF-β: MAB 240, BAF 240, 240-B). Concentration of IL-10 in cell culture supernatants was determined by DUO SET DY217B (R&D Systems, Minneapolis, MN, USA) according to the manufacturer’s instructions.

#### 2.3.2 Detection of immunoglobulin A antibodies against *Escherichia coli*

Antibodies specific against *E. coli* were determined in sera and stool of supplemented and non-supplemented children by ELISA. Wells of 96-well plates were coated by heat-inactivated EcO83 (diluted in phosphate buffered saline (PBS) to concentration 10^8^ CFU/ml, 100 μl/well). Sera and stool samples were diluted 10× and 100×, respectively. Biotinylated antibody against IgA (purchased from Sevapharma, diluted 500×) was added. After two washings by PBS followed by additional two washings by PBS with 0.05% Tween, substrate (o-phenylenediamine) was added in each well, the reaction was stopped by 50 μl of 2 M sulfuric acid, and optical density was read at wavelength 492 nm. The mixture of normal human sera was used as a calibrator to determine the concentration of IgA.

#### 2.3.3 Detection of immunoglobulin E specific against food and air allergens

IgE specific against mixtures of air allergens (DYNX 1) and food allergens (FX 04) was detected in sera of the peripheral blood of 10-year-old children by ELISA according to the manufacturer’s recommendation (Dr. Fooke Laboratorien).

### 2.4 Flow cytometry analyses of the proportion of regulatory T cells, presence of immunoregulatory molecules on regulatory T cells, proportion and maturational status of myeloid dendritic cells and plasmacytoid dendritic cells in peripheral blood

For characterization of cell surface markers on Treg, peripheral blood was stained against CD4 (fluorescein isothiocyanat (FITC), clone RPA-T4; BD, Franklin Lakes, NJ, USA), CD25 (PerCP-Cy5.5, clone MEM181; Exbio, plc, Vestec, Czechia), CD127 (PE-Cy7, clone A019D5), CTLA-4 (APC, clone L3D10), PD-1 (APC-Cy7, clone EH12.2H7), and GITR (PE, clone 621), all from BioLegend, San Diego, CA, USA. To evaluate the proportion of particular DC subsets and their maturational status, the peripheral blood of 10-year-old children was stained by antibodies against CD11c conjugated with APC (130-113-584, clone REA618; Miltenyi Biotec, Auburn, CA, USA), CD123 conjugated with FITC (1F-700-T100, clone 6H6) and CD83 (1P-677-T100, clone HB15e), both purchased from Exbio, plc (Vestec, Czechia). After 15-min incubation, red blood cells were lysed using BD FACS lysing solution (349202; BD, Franklin Lakes, NJ, USA), washed three times with PBS, and acquired immediately using BD FACS Canto II (Franklin Lakes, NJ, USA). The proportion of Treg was determined using TregFlowEx Kit, 7417 Exbio, plc. The kit contains all reagents including antibodies against cell surface markers (CD4 FITC, MEM-241; CD25 PE, clone MEM-181) and permeabilization/fixation solutions for Treg determination including antibody against transcription factor FoxP3 (APC, clone 3G3). Flow cytometry data were analyzed using FlowJo software v7.2 (Franklin Lakes, NJ, USA).

### 2.5 Isolation of myeloid dendritic cells and plasmacytoid dendritic cells from umbilical cord blood

Cord blood was collected from 47 donors, and cord blood mononuclear cells (CBMCs) were isolated as previously described (43, 45). Briefly, after signed written informed consent, approximately 30 ml of cord blood was collected. CBMCs were isolated by gradient centrifugation. mDCs and pDCs were isolated from CBMCs using Myeloid Dendritic Cell Isolation Kit, human, 130-094-487, and CD303 (BDCA-2) MicroBead Kit, human, 130-090-509, respectively (both purchased from Miltenyi Biotec, Auburn, CA, USA).

### 2.6 Generation of myeloid dendritic cells and plasmacytoid dendritic cells from progenitor cells

mDCs were generated from the adherent fraction of CBMCs in the presence of recombinant human growth factors recombinant human Granulocyte Macrophage Colony Stimulating Factor (rhGM-CSF) (300–03) and rhIL-4 (200-04) purchased from PeproTech (Rocky Hill, NJ, USA) as previously described (45). pDCs were obtained from CD34+ progenitor cells isolated from CBMCs using CD34 MicroBead Kit, human, 130-046-703 (Miltenyi Biotec, Auburn, CA, USA), after 7 days of cultivation in the presence of growth factors rhIL-3 (200-03) and rhFlt3L (300-19) PeproTech (Rocky Hill, NJ, USA) in an incubator with regulated CO_2_ atmosphere (5.5% CO_2_) at 37°C.

### 2.7 Stimulation of myeloid dendritic cells and plasmacytoid dendritic cells by Escherichia coli O83:K24:H31

The impact of EcO83 on the maturational status of cord blood DCs was tested on both directly isolated and *in vitro*-generated mDCs and pDCs. DCs were stimulated by EcO83 in the ratio 10 bacterial cells:1 DC *in vitro*. The maturational state of DCs was checked by flow cytometry after 24 h of stimulation, as described for peripheral blood above. Due to the limited volume of cord blood, we were not able to perform parallel analyses of mDCs, monocyte-derived dendritic cells (moDCs), and pDCs from the same donor.

### 2.8 Gut microbiota analyses

DNA was isolated from rectal swabs using QIAmp Fast DNA Stool Mini Kit (cat. no. 51604; Qiagen) according to the manufacturer’s recommendation. The total extracted DNA was used for high-throughput sequencing of the V3-V4 region of the 16S rRNA gene. The amplification reaction was performed with the set of specific primers with barcodes (341F GTCCTACGGGNGGCWGCAG and 806R GGACTACHVGGGTWTCTAAT) using HiFi HotStart Ready Mix (Roche) as follows: initial denaturation step 3 min at 95°C followed by 25 cycles at 95°C for 30 s, 55°C for 30 s, 72°C for 30 s with a final elongation step at 72°C for 5 min using 12.5 ng of DNA. Besides the isolated DNA, ZymoBIOMICS Microbial Community Standard and Standard II (log distribution), as well as ZymoBIOMICS Microbial Community DNA Standard and Standard II (log distribution), were used to assess the performance of entire metagenomic workflows (Zymo Research). PCR products were checked using QIAxcel advanced capillary electrophoresis (QIAgen, Hilden, Germany). Triplicates of the amplicons were pooled, normalized with the SequalPrep™ Normalization Plate Kit (ThermoFisher Scientific), and concentrated on a Concentrator 5301 (Eppendorf) for approximately 3 h at 30°C under vacuum. The resulting volume was purified using the DNA Clean & Concentrator kit (Zymo Research). The amplicon library was then ligated with sequencing adapters (TruSeq DNA PCR-free LT Sample Preparation Kit, Illumina) using KAPA HyperPlus Kit (Roche), pooled in equimolar concentrations, and sequenced. Amplicon sequencing was performed with the Miseq platform (Illumina). The sequencing data have been uploaded to the online repository and are available at the Sequence Read Archive under accession number PRJNA886861.

### 2.9 Statistics

The comparison of allergy incidence in children among the groups was evaluated using statistical methodology for recurrent time-to-event data. The within-person variability was modeled by involving a frailty parameter in the parametric or semiparametric class of models for survival data. The latter class was represented by the Gaussian and Gamma frailty version of the Cox proportional hazards model, while the former included the Gaussian and Gamma frailty Weibull survival model. The Akaike Information Criterion (AIC) was used to determine the most appropriate model for interpreting the findings. The lower values of AIC indicate the more plausible model. Three cohorts of newborn babies were followed prospectively in time, and the allergy status was determined using scheduled visits with the allergist. When scheduled visits with the allergist could not be completed, parent assessment was pursued instead. Statistical analyses are provided for (i) the case of allergy incidence being confirmed exclusively by the allergist and (ii) allergy incidence ascertained by the allergist or parental report. These results are summarized in **Tables 1A, B**. Differences between the groups of healthy and allergic children were evaluated using the unpaired t-test for normally distributed data [proportion of CD83 mDCs and pDCs in the peripheral blood of 10-year-old children, median fluorescence intensity (MFI) of CD83]. ANOVA was employed for statistical evaluation of data with normal distribution when comparing more than two of the groups (proportion of Treg and percentages of markers associated with functional capacity of Treg, proportion of CD83-positive mDCs and pDCs in the peripheral blood of 10-year-old children, MFI of CD83, proportion of CD83-positive mDCs, moDCs, and pDCs stimulated with EcO83 *in vitro*). Simultaneous tests for general linear hypotheses, preserving the Type 1 error rate under multiple comparisons, were used in analyzing the microbiota. Otherwise, Kruskal–Wallis test was used. To prevent false positivity caused by many simultaneous tests at the same time, Bonferroni *post-hoc* tests (GraphPad Prism software, GraphPad Software, San Diego, CA, USA) were employed. The results are expressed as mean ± standard error of the mean for data with normal distribution. Data without normal distribution are presented as median with standard deviation. Statistical significance was set at p ≤ 0.05.

**Table 1:** Allergy incidence in 10-year-old children.

TABLE 1A Allergy incidence confirmed by an allergist.					
Model	Covariate	Hazard ratio (HR)	95% Confidence limits for HR	p-value	AIC
Gaussian-frailty Cox PH	Male Gender	1.9390	(1.3159, 2.8569)	0.0008	1,429.753
	Group S AM	0.7846	(0.5244, 1.1738)	0.2379	
	Group N NM	0.5534	(0.3315, 0.9237)	0.0236	
Gamma-frailty Cox PH	Male Gender	1.9689	(1.3387, 2.8956)	0.0006	1,442.232
	Group S AM	0.7834	(0.5252, 1.1685)	0.2315	
	Group N NM	0.5482	(0.3291, 0.9131)	0.0209	
Gaussian-frailty Weibull PH	Male Gender	1.7383	(1.1538, 2.6188)	0.0082	2,778.740
	Group S AM	0.6568	(0.4309, 1.0011)	0.0506	
	Group N NM	0.6505	(0.3831, 1.1046)	0.1114	
Gamma-frailty Weibull PH	Male Gender	1.7105	(1.1337, 2.5806)	0.0105	2,770.265
	Group S AM	0.6721	(0.4406, 1.0252)	0.0651	
	Group N NM	0.6748	(0.3956, 1.1510)	0.1488	
TABLE 1B Allergy incidence in children confirmed by an allergist and/or parental report.					
Model	Covariate	Hazard ratio (HR)	95% Confidence limits for HR	p-value	AIC
Gaussian-frailty Cox PH	Male Gender	1.7379	(1.2444, 2.4270)	0.0012	1,872.181
	Group S AM	0.7007	(0.4940, 0.9937)	0.0460	
	Group N NM	0.4230	(0.2622, 0.6826)	0.0004	
Gamma-frailty Cox PH	Male Gender	1.7547	(1. 2581, 2.4473)	0.0009	1,884.426
	Group S AM	0.6930	(0.4895, 0.9811)	0.0387	
	Group N NM	0.4148	(0.2574, 0.6685)	0.0003	
Gaussian-frailty Weibull PH	Male Gender	1.6782	(1.1851, 2.3766)	0.0035	3,488.320
	Group S AM	0.6494	(0.4513, 0.9345)	0.0201	
	Group N NM	0.5416	(0.3286, 0.8929)	0.0162	
Gamma-frailty Weibull PH	Male Gender	1.6558	(1.1689, 2.3457)	0.0045	3,480.683
	Group S AM	0.6656	(0.4625, 0.9578)	0.0284	
	Group N NM	0.5627	(0.3404, 0.9303)	0.0250	

S AM, E. coli O83:K24:H31-supplemented children of allergic mothers.

N NM, non-supplemented children of non-allergic mothers.

AIC - Akaike Information Criterion, PH - proportional hazard (model).

## 3 Results

### 3.1 Early postnatal Escherichia coli O83:K24:H31 supplementation decreased allergy incidence in 10-year-old children

Early postnatal supplementation of newborns with EcO83 has been described to lower allergy incidence in children. In the current report, the allergy status of 10-year-old children was inspected by an allergist to confirm that the early postnatal EcO83 supplementation limits the development of allergic diseases (41). Parental reports of possible clinical signs of allergy manifestation were confirmed by specific IgE against allergens and/or positive skin prick test. Specific allergy of children in the current cohort is provided in **Supplementary Table S2**. In the group of N HM, four children out of 23 were allergic at the age of 10 years. In the group of N AM, 13 children out of 38 were allergic, and finally, in the group of EcO83-supplemented children, nine children out of 45 were allergic.

EcO83 supplementation lowered the allergy incidence in the high-risk group for allergy development (children of AMs) to a level comparable to that of the group of low-risk children (children of healthy mothers). The comparison of the incidence of allergy among the groups based on allergist confirmation of allergy is reported in **Table 1A**. To increase the number of children inspected, we have added children whose allergy status was determined only by telephonic contact with their parents at the age of 10 years (**Table 1B**). Surprisingly, the most striking difference in allergy incidence was between boys and girls. Allergy incidence in boys was approximately double in comparison with that in girls (**Table 1**).

When comparing the allergy incidence confirmed only by the allergist, allergy incidence tends to be lower in S AM compared to N AM using Weibull proportional hazards models (PH model).

After additional inclusion of the children with allergy status reported by parents, allergy incidence was significantly lower in S AM compared to N AM using different statistical models (**Table 1**). In the group of N HM, six children out of 32 were allergic at the age of 10 years. In the group of N AM, 16 children out of 42 were allergic, and finally, in the group of EcO83-supplemented children, 13 children out of 52 were allergic.

Not surprisingly, allergy incidence in the low-risk group (N HM) was lower than that in the group of children of AMs.

### 3.2 Impact of Escherichia coli O83:K24:H31 supplementation on cytokine, immunoglobulin A, and immunoglobulin E concentrations in peripheral blood of 10-year-old children

#### 3.2.1 Dynamics of cytokine levels in sera

**Figure 1:**
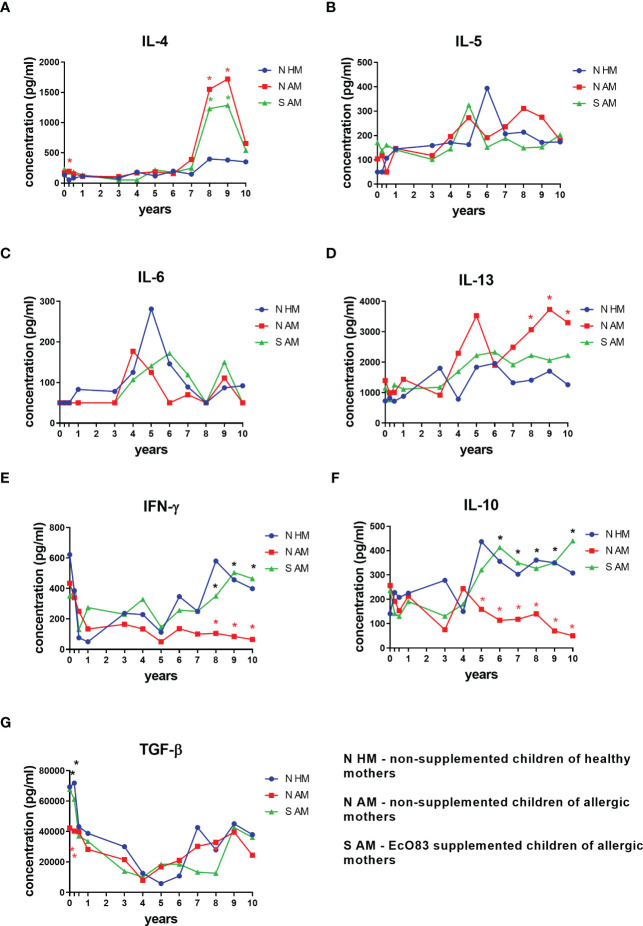
Cytokine concentration in cord and peripheral blood sera of children until the age of 10 years. Cytokines were determined by ELISA. Typical Th2 cytokines were determined: IL-4 **(A)**, IL-5 **(B)**, IL-6 **(C)**, and IL-13 **(D)**. The concentration of typical Th1 cytokine (IFN-γ) is shown in **(E)** Dynamics of concentration of immunoregulatory cytokines is presented in **(F)** for IL-10 and panel **(G)** for TGF-β. EcO83, *Escherichia coli* O83:K24:H31; *p ≤ 0.0071 (adjusted p value). IL, interleukin; IFN, interferon; TGF, transforming growth factor.

The effect of early postnatal supplementation of newborns with EcO83 on the dynamics of cytokines in their peripheral blood has been followed. Here, we are presenting results from birth (cord blood) until the age of 10 years. At the age of 3 months, a significantly elevated concentration of IL-4 in the sera of N AM was observed in comparison with that in the children of healthy mothers. Cytokine concentrations exhibit dynamic changes over childhood development as well as marked interindividual variability. Nevertheless, elevated concentrations of IL-4 were detected in the sera of both N AM and S AM at the ages of 8 and 9 years. No impact of EcO83 supplementation on lowering of this typical Th2 cytokine has been observed until the age 10 years (**Figure 1A**). The other typical Th2 cytokine IL-5 showed a trend of increasing values during ontogenesis, although there was no statistical significance among the three main groups under study. The increased concentration of IL-5 in N AM at the ages of 8 and 9 years has not reached statistical significance (**Figure 1B**). The concentration of IL-6 is quite dynamic, but no significant impact of EcO83 supplementation has been recorded (**Figure 1C**). The last Th2 cytokine tested, IL-13, showed increased concentration in N AM in comparison with that in N HM. No effect of EcO83 supplementation on the level of IL-13 has been documented (**Figure 1D**).

IFN-γ was selected as a typical Th1 cytokine in our study. There is a significant drop in the concentration of IFN-γ after the delivery, and the levels of IFN-γ remain quite low in the group of N AM in comparison with those in both N HM and S AM. Significantly lower levels of IFN-γ in the group of N AM in comparison with those in N HM and S AM were detected at the ages of 8, 9, and 10 years (**Figure 1E**).

The impact of early postnatal EcO83 administration on the concentration of immunoregulatory cytokines IL-10 and TGF-β was followed. Significantly decreased levels of IL-10 in the sera of N AM in comparison with those in N HM were determined at the ages of 5, 6, 7, 8, 9, and 10 years. Interestingly, EcO83 supplementation increased concentration of IL-10 in the group of S AM in comparison with N AM at the ages of 6, 7, 8, 9, and 10 years (**Figure 1F**). This important capacity of EcO83 to promote IL-10 secretion could contribute to the observed lower allergy incidence in the group of S AM. TGF-β plays an important role in isotype switching to IgA (46, 47), which is generally impaired in children until the age of 15 years compared with adults (48, 49). Therefore, the lower concentration of TGF-β in cord blood with a significant drop in the concentration of TGF-β during the postnatal period could be associated with a lower IgA production, possibly predisposing the children to an increased risk of infection. This decrease in TGF-β concentration is more pronounced in the group of N AM in comparison with both N HM and S AM at the age of 3 months (**Figure 1G**). Our data indicate that early postnatal supplementation of newborns with EcO83 has only a marginal effect on the cytokine concentration in the peripheral blood of children during early infancy. Rather, the effect of maternal allergy status contributes to the observed differences in early postnatal cytokine concentrations among groups. EcO83 was not able to downregulate the concentration of Th2 cytokines. Only the immunoregulatory cytokine IL-10 and Th1 cytokine IFN-γ were increased in the group of EcO83-supplemented children compared with N AM at the ages of 6, 7, 8, 9, 10 years and 8, 9, 10 years, respectively.

The individual levels of cytokine concentration in the sera of children divided according to their allergy status at the age of 7 until 10 years are presented in **Supplementary Figure S1**. This figure highlights high individual variability. After subdivision of the three basic groups of children into the six subgroups, only changes in some cytokines remain significantly different. Levels of IL-4 in the sera of both non-allergic non-supplemented children of allergic mothers (H N AM) and non-allergic supplemented children of allergic mothers (H S AM) were significantly higher compared with those in non-allergic non-supplemented children of healthy mothers (H N HM) (**Supplementary Figure S1A**). No significant difference in cytokine levels among particular groups was observed for IL-5 and IL-6 (**Supplementary Figures S1B, S1C**, respectively). The concentration of IL-13 of allergic non-supplemented children of healthy mothers (A N AM) was significantly increased in comparison with that in A N HM (**Supplementary Figure S1D**). Levels of IFN-γ were higher in H N HM and H S AM compared with that in H N AM. H N HM and H S AM have significantly increased concentrations of IFN-γ compared with those in A N HM and allergic EcO83-supplemented children of allergic mothers (A S AM), respectively (**Supplementary Figure S1E**). Both groups of H N HM and H S AM have increased levels of immunoregulatory cytokine IL-10 compared with that in H N AM (**Supplementary Figure S1F**). No significant change in blood levels of TGF-β has been detected (**Supplementary Figure S1G**).

#### 3.2.2 The impact of early postnatal Escherichia coli O83:K24:H31 supplementation on immunoglobulin A secretion

We have detected the concentration of IgA antibodies against *E. coli* in both sera and stool samples of non-supplemented and EcO83-supplemented children from day 3 to 3 years. The concentration of IgA specific against *E. coli* in sera did not differ among supplemented and non-supplemented children at the age of 3 days (**Supplementary Figure S2A**). At the age of 3 months, there is a significant increase of IgA in S AM compared to both N HM and N AM (**Supplementary Figure S2B**). Similarly, EcO83 supplementation promoted *E. coli*-specific IgA secretion at the age of 6 months (**Supplementary Figure S2C**), 12 months (**Supplementary Figure S2D**), 24 months (**Supplementary Figure S2E**), and 36 months (**Supplementary Figure S2F**).

The production of secretory IgA specific against *E. coli* was detected in stool samples. Not surprisingly, no difference was found at the age of 3 days (**Supplementary Figure S3A**). EcO83 supplementation promoted IgA levels in stool samples at the age of 3 months (**Supplementary Figure S3B**). The capacity of EcO83 to elevate IgA production was still evident at the age of 6 months (**Supplementary Figure S3C**), 12 months (**Supplementary Figure S3D**), and 24 months (**Supplementary Figure S3E**). We were not able to detect a significantly different concentration of IgA among supplemented and non-supplemented children at the age of 36 months (**Supplementary Figure S3F**).

#### 3.2.3 Concentration of allergen-specific immunoglobulin E

The levels of allergen-specific IgE were followed in 10-year-old children. No significant difference in concentrations of IgE specific against the mixture of food allergens was observed among the three basic groups (**Supplementary Figure S4A**), among the six subgroups of children divided according to their allergy at the age of 10 years (**Supplementary Figure S4B**), or when the children were divided only according to their own allergy status regardless of allergy status of their mother or possible EcO83 supplementation (**Supplementary Figure S4C**). Similarly, no significant difference in the concentration of IgE specific against the mixture of respiratory allergens was observed (**Supplementary Figure S4D**). After subdivision of the three basic groups according to the allergy at the age of 10 years, significantly increased levels of IgE specific against respiratory allergens were detected in A N HM, A N AM, and A S AM compared with H N AM, H N AM, and H S AM, respectively (**Supplementary Figure S4E**). Children suffering from allergy against some of the respiratory allergens had elevated levels of IgE compared with non-allergic ones (**Supplementary Figure S4F**).

### 3.3 Proportion of regulatory T cells and cell surface presence of markers associated with the functional capacity of regulatory T cells

**Figure 2:**
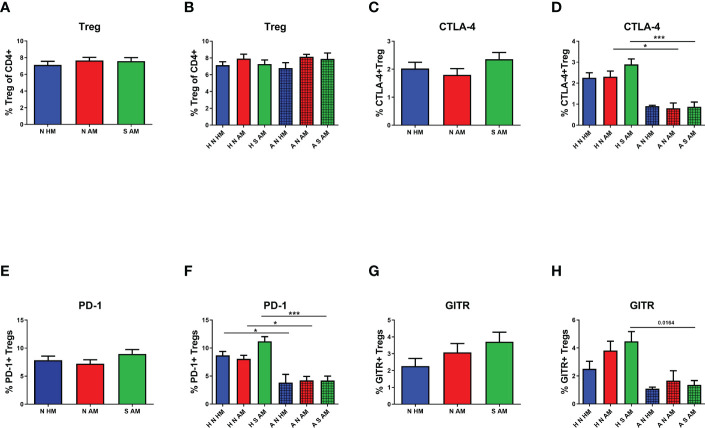
The proportion and functional characteristics of Treg in the peripheral blood of 10-year-old children were determined by flow cytometry. The percentage of Treg in the peripheral blood in the three basic groups is shown in **(A)** Percentages of Treg in children divided according to their own as well as maternal allergy status and appropriate probiotic supplementation are presented in **(B)** The proportion of Treg positive for cytotoxic T-lymphocyte antigen 4 (CTLA-4) in the three basic groups is indicated in **(C)** CTLA-4-positive Treg in children divided according to their own as well as maternal allergy status and appropriate probiotic supplementation are presented in **(D)** The presence of programmed death domain 1 (PD-1) on Treg of children divided into the three basic groups **(E)** and the six subgroups **(F)**. The cell surface presence of glucocorticoid-induced tumor necrosis factor receptor-related gene (GITR) on Treg in children divided into the three basic groups is indicated in **(G)** and the six subgroups in **(H)** N HM, non-supplemented children of healthy mothers; N AM, non-supplemented children of allergic mothers; S AM, *E coli* O83:K24:H31-supplemented children of allergic mothers; H N HM, healthy non-supplemented children of healthy mothers; H N AM, healthy non-supplemented children of allergic mothers; H S AM, healthy *E coli* O83:K24:H31-supplemented children of allergic mothers; A N HM, allergic non-supplemented children of healthy mothers; A N AM, allergic non-supplemented children of allergic mothers; A S AM, allergic *E coli* O83:K24:H31-supplemented children of allergic mothers; Treg, regulatory T cells; *p ≤ 0.0125 (adjusted p value); ***p ≤ 0.00025 (adjusted p value).

To evaluate the possible effect of EcO83 supplementation on the proportional and functional characteristics of Treg, Treg were inspected in the peripheral blood of 10-year-old children.There was no significant difference in the proportion of Treg among the three basic groups (**Figure 2A**). After division of children into subgroups based on their allergy status at the age of 10 years, no statistically significant results were obtained either (**Figure 2B**). The markers pointing to the functional capacity of Treg were followed by flow cytometry as well. Cell surface presence of cytotoxic T-lymphocyte antigen 4 (CTLA-4) on Treg was not statistically different when compared among the three basic groups (**Figure 2C**). After subdivision of these three basic groups according to the children’s allergy status at the age of 10 years, no significantly different cell surface presence of CTLA-4 was detected in the children of healthy mothers. Lower CTLA-4 was observed in allergic non-supplemented children of allergic mothers (A N AM) in comparison with H N AM (p = 0.008). Similarly, decreased presence of CTLA-4 has been observed in A S AM in comparison with that in H S AM (p = 0.0001) (**Figure 2D**). The programmed death domain 1 (PD-1) was followed on Treg as another marker pointing to the functional capacity of Treg. No significant difference among N HM, N AM, and S AM has been observed (**Figure 2E**). When the children were divided according to allergy status, significantly lower levels of PD-1 were detected in the group of A N HM in comparison with those in H N HM (p = 0.008). Decreased presence of PD-1 was ascertained in A N AM compared with that in H N AM (p = 0.004). PD-1 was lower in the group of A S AM in comparison with that in H S AM (p = 0.0001) (**Figure 2F**). Finally, glucocorticoid-induced tumor necrosis factor receptor-related gene (GITR) was tested without a significant difference among the three basic groups and six subgroups of children divided according to the allergy status at the age of 10 years (**Figures 2G, H**). Neither the proportion nor the markers associated with functional capacity of Treg were affected by early postnatal supplementation of neonates with EcO83. We have described impaired functional capacity of Treg of allergic children compared with non-allergic children.

### 3.4 Maturational status of myeloid dendritic cells and plasmacytoid dendritic cells in peripheral blood of 10-year-old children

**Figure 3:**
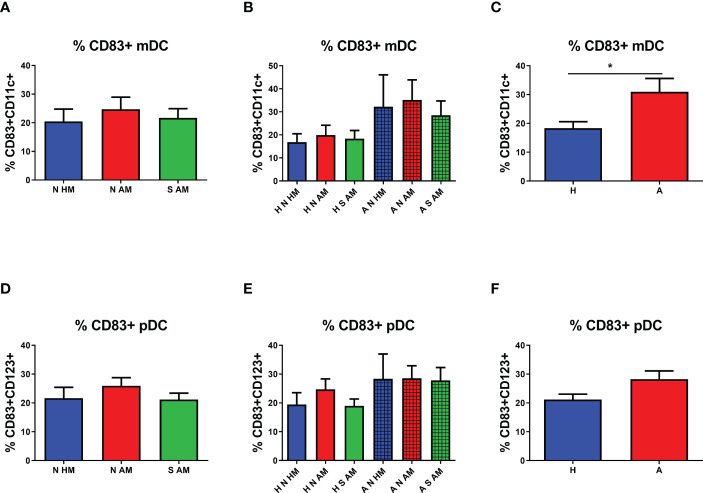
Proportion of CD83+ myeloid dendritic cells (mDCs) and plasmacytoid dendritic cells (pDCs) in the peripheral blood of 10-year-old children detected by flow cytometry. The presence of CD83 on mDCs of allergic non-supplemented children of healthy mothers (A N HM), allergic non-supplemented children of allergic mothers (A N AM), and allergic *E coli* O83:K24:H31-supplemented children of allergic mothers (A S AM) is documented in **(A)** The proportion of CD83+ mDCs in the peripheral blood of children divided according to their allergic status into the six subgroups is shown in **(B)** Levels of CD83 on mDCs of children divided only according to their allergic status are presented in **(C)** The proportion of CD83+ pDCs of N HM, N AM, and S AM is documented in **(D)** Numbers of CD83+ pDCs in the peripheral blood of children divided according to their allergic status into the six subgroups are shown in **(E)** The percentage of CD83 on mDCs of children divided only according to their allergic status are presented in **(F)** H N HM, healthy non-supplemented children of healthy mothers; H N AM, healthy non-supplemented children of allergic mothers; H S AM, healthy *E coli* O83:K24:H31-supplemented children of allergic mothers; A N HM, allergic non-supplemented children of healthy mothers; A N AM, allergic non-supplemented children of allergic mothers; A S AM, allergic *E coli* O83:K24:H31-supplemented children of allergic mothers; *p ≤ 0.0167 (adjusted p value). H, healthy children regardless of maternal allergy status or Escherichia coli O83:K24:H31 supplementation; A, allergic children regardless of maternal allergy status or Escherichia coli O83:K24:H31 supplementation.

The possible effect of EcO83 supplementation on functional characteristics of the two most abundant DC subsets was analyzed by flow cytometry. In our previous study, CD83 has been identified as a marker with the highest sensitivity to stimulation (45). Therefore, the cell surface presence of CD83 has been inspected on both mDCs and pDCs. No significant difference in the proportion of CD83+ mDCs in the peripheral blood of 10-year-old children was detected among the three basic groups (**Figure 3A**). After subdivision of the three basic groups according to the allergy status, no statistically significant differences in the cell surface presence of CD83 was observed (**Figure 3B**). When the proportion of CD83+ mDCs was compared in healthy and allergic children regardless of their maternal allergy status and eventual EcO83 supplementation, an elevated presence of CD83 was measured on mDCs of allergic children in comparison with that in non-allergic ones (p = 0.0067) (**Figure 3C**). The cell surface presence of CD83 on pDCs was not different among the three basic groups or the six subgroups of children (**Figures 3D, E**), respectively. When the cell surface presence of CD83 was analyzed on pDCs in children divided into two groups based on their allergy status, no significant difference in the cell surface presence was detected on pDCs of children suffering from allergy either (**Figure 3F**).

**Figure 4:**
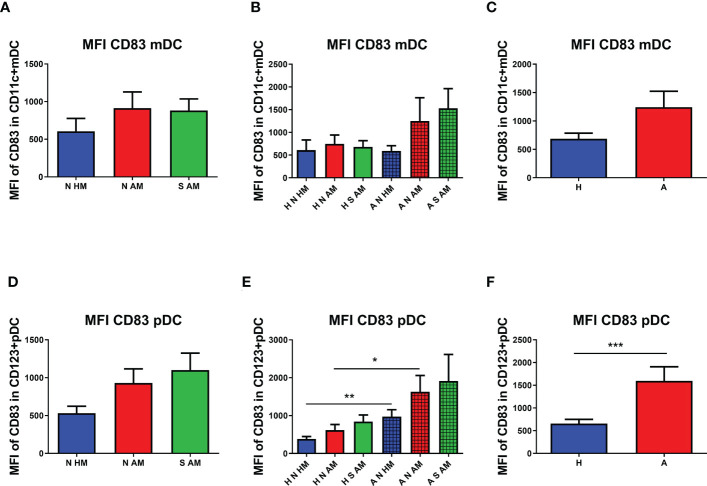
Median fluorescence intensity (MFI) of CD83 on myeloid dendritic cells (mDCs) and plasmacytoid dendritic cells (pDCs). The MFI of CD83 on mDCs of allergic non-supplemented children of healthy mothers (A N HM), allergic non-supplemented children of allergic mothers (A N AM), and allergic *E coli* O83:K24:H31-supplemented children of allergic mothers (A S AM) is presented in **(A)** The MFI of CD83+ mDCs in the peripheral blood of children divided according to their allergic status into the six subgroups is documented in **(B)** Levels of the MFI of CD83 on mDCs of children divided only according to their allergic status are presented in **(C)** The MFI of CD83+ pDCs of N HM, N AM, and S AM is documented in **(D)** The MFI of CD83+ pDCs in the peripheral blood of children divided according to their allergic status into the six subgroups is shown in **(E)** The MFI of CD83 on pDCs of children divided only according to their allergic status is presented in **(F)** A N HM, allergic non-supplemented children of healthy mothers; A N AM, allergic non-supplemented children of allergic mothers; A S AM, allergic *E coli* O83:K24:H31-supplemented children of allergic mothers; *p ≤ 0.00167 (adjusted p value); **p ≤ 0.0033 (adjusted p value); ***p ≤ 0.0003 (adjusted p value). H, healthy children regardless of maternal allergy status or Escherichia coli O83:K24:H31 supplementation; A, allergic children regardless of maternal allergy status or Escherichia coli O83:K24:H31 supplementation.

To quantify the presence of CD83 on DCs, the MFI was employed. No statistically significant difference in the MFI of CD83 on mDCs in the peripheral blood of 10-year-old children was observed (**Figure 4A**). After subdivision of children according to their allergy status, non-significant values of the MFI of CD83 on mDCs of A N AM and A S AM were ascertained (**Figure 4B**). When the MFI of CD83 was compared in children sorted according to their allergy status, no change in the MFI of CD83 was observed on mDCs of allergic children in comparison with non-allergic ones (**Figure 4C**). The MFI of CD83 was tested on pDCs as well. No difference in the MFI of CD83 on pDCs among the three basic groups was determined (**Figure 4D**). After division of the children according to their allergy status at the age of 10 years, a general trend to increased values of the MFI in allergic children is quite evident. The MFI of CD83 on pDCs of A N HM was significantly increased compared with the MFI of CD83 in H N HM (p = 0.0017). Similarly, the MFI of CD83 on pDCs of A N AM was significantly increased compared with the MFI of CD83 in H N AM (p = 0.0093). Finally, the MFI of CD83 on pDCs of A S AM was not statistically different from the MFI of CD83 in H S AM (**Figure 4E**). Next, the MFI of CD83 was evaluated in children divided into two groups based only on their allergy status. A higher MFI of CD83 on pDCs of allergic children was documented in comparison with that in non-allergic ones (p = 0.0002) (**Figure 4F**). Our data do not support the hypothesis that early postnatal EcO83 supplementation affected long-term functional characteristics of mDCs and/or pDCs. On the other hand, increased functional capacity of mDCs was described in the group of allergic children compared with that in non-allergic ones, suggesting increased reactivity of these DCs. Therefore, more reactive DCs of allergic children could initiate inappropriate immune responses to relatively innocuous allergens upon allergen encounter.

### 3.5 The effect of Escherichia coli O83:K24:H31 on maturational status of myeloid dendritic cells, monocyte-derived dendritic cells, and plasmacytoid dendritic cells *in vitro*

**Figure 5:**
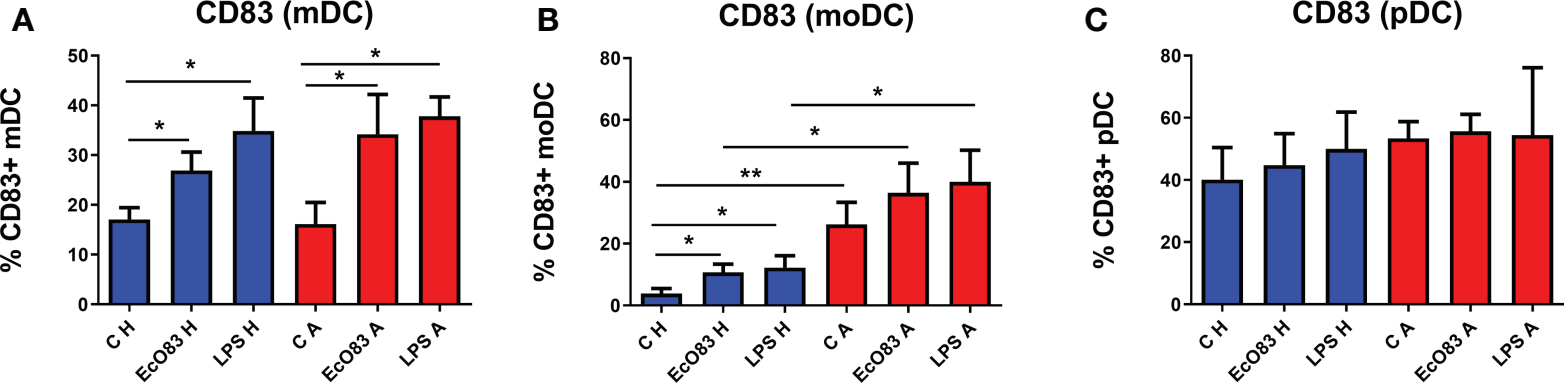
The effect of *E coli* O83:K24:H31 (EcO83) on the induction of the activation marker CD83 on myeloid dendritic cells (mDCs), monocyte-derived dendritic cells (moDCs), and plasmacytoid dendritic cells (pDCs) obtained from the cord blood of newborns of healthy and allergic mothers. mDCs isolated directly from cord blood cells were stimulated with EcO83, and the cell surface activation marker CD83 was inspected after 24 h **(A)**. The effect of EcO83 stimulation on the maturation of moDCs is presented in **(B)** The impact of EcO83 stimulation on the induction of CD83 on *in vitro*-derived pDCs is shown in **(C)** C H, non-stimulated DCs of newborns of healthy mothers; C A, non-stimulated DCs of newborns of allergic mothers; EcO83 H, DCs of newborns of healthy mothers stimulated with *E coli* O83:K24:H31; EcO83 A, DCs of newborns of allergic mothers stimulated with *E coli* O83:K24:H31; LPS H, DCs of newborns of healthy mothers stimulated with lipopolysaccharide; LPS A, DCs of newborns of allergic mothers stimulated with lipopolysaccharide; *p ≤ 0.05; **p ≤ 0.01.

To evaluate the capacity of EcO83 to promote the maturation of the neonatal immune system, mDCs were isolated directly from cord blood. We were able to obtain only small amounts of pDCs directly from cord blood; therefore, pDCs were generated from CD34+ cord blood precursor cells. To ascertain whether there is a difference between mDCs isolated directly from cord blood and moDCs generated from the adherent fraction of CBMCs, both mDCs and moDCs were stimulated by EcO83. EcO83 was able to promote the maturation of mDCs from cord blood of children of healthy mothers (p = 0.0380). Similarly, EcO83 promoted the maturation of mDCs of newborns of AMs (p = 0.0142). LPS was used as a positive control only in some experiments, as we were not always able to obtain sufficient amounts of mDCs. LPS promoted the maturation of mDCs of children of both healthy mothers (p = 0.0234) and allergic mothers (p = 0.0336) (**Figure 5A**).

EcO83 promoted the maturation of moDCs derived from cord blood precursor cells of newborns of healthy mothers (p = 0.0106). LPS stimulation promoted the maturation of moDCs of newborns of healthy mothers as well (p = 0.0142). The percentage of CD83-positive non-stimulated moDCs of newborns of AMs was significantly elevated in comparison with non-stimulated moDCs of newborns of healthy mothers (p = 0.0078). Both EcO83 and LPS stimulation promoted the maturation of moDCs of neonates of AMs, although the difference was not statistically significant. On the other hand, the percentage of CD83-positive moDCs of newborns of AMs was increased after EcO83 and LPS stimulation in comparison with non-allergic ones (p = 0.0424 and p = 0.0244, respectively) (**Figure 5B**).

pDCs have been shown to play a role in asthma (50). To test whether EcO83 can affect pDCs, *in vitro*-derived pDCs were stimulated by EcO83, and the cell surface activation marker CD83 was inspected by flow cytometry. Surprisingly, neither EcO83 stimulation nor maternal allergy status had an effect on the cell surface presence of CD83 on pDCs (**Figure 5C**). We have shown that EcO83 is able to promote the maturation of both mDCs and moDCs without any impact on pDCs. Interestingly, only moDCs from the cord blood of newborns of AMs exerted increased reactivity in comparison with mDCs directly isolated from cord blood, challenging the biological relevance of results obtained from *in vitro*-derived DCs.

### 3.6 Escherichia coli O83:K24:H31 promotes interleukin 10 secretion in myeloid dendritic cells, monocyte-derived dendritic cells, and plasmacytoid dendritic cells

**Figure 6:**
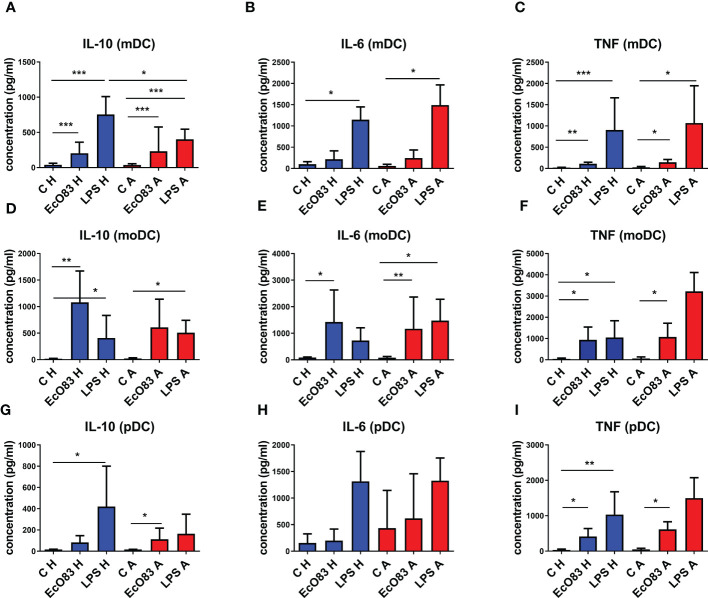
The impact of *E. coli* O83:K24:H31 (EcO83) stimulation on cytokine secretion by dendritic cells (DCs), as determined by ELISA. The capacity of EcO83 to promote IL-10 secretion by myeloid dendritic cells (mDCs) is shown in **(A)**. The impact of EcO83 on the production of pro-inflammatory cytokines IL-6 and TNF is shown in **(B, C)**, respectively. The capacity of EcO83 to promote IL-10 secretion by monocyte-derived dendritic cells (moDCs) is shown in **(D)**. Release of IL-6 and TNF by moDC is presented in (**E**, **F**, respectively. The effect of EcO83 stimulation on the secretion of IL-10 by pDCs is documented in **(G)**. Concentration of pro-inflammatory cytokines IL-6 and TNF in cell culture supernatants of pDC stimulated with EcO83 and LPS is indicated in **(H, I)**. C H, non-stimulated DCs of newborns of healthy mothers; C A, non-stimulated DCs of newborns of allergic mothers; EcO83 H, DCs of newborns of healthy mothers stimulated with *E. coli* O83:K24:H31; EcO83 A, DCs of newborns of allergic mothers stimulated with *E. coli* O83:K24:H31; LPS H, DCs of newborns of healthy mothers stimulated with lipopolysaccharide; LPS A, DCs of newborns of allergic mothers stimulated with lipopolysaccharide; *p ≤ 0.0167 (adjusted p value); **p ≤ 0.0033 (adjusted p value); ***p ≤ 0.00033 (adjusted p value). IL, interleukin; TNF, tumor necrosis factor.

The key role of IL-10 in promoting immunoregulatory responses has been acknowledged, and DCs can represent an important source of this cytokine. Therefore, the possible capacity of EcO83 to induce IL-10 expression and secretion by DCs was tested. EcO83 was able to promote IL-10 secretion by mDCs of newborns of both healthy (p = 0.0001) and allergic (p = 0.0001) mothers. As expected, LPS stimulated IL-10 secretion in mDCs of newborns of both healthy (p = 0.0001) and allergic (p = 0.0001) mothers. IL-10 concentration in cell culture supernatants of LPS-stimulated mDCs of newborns of healthy mothers was higher compared to that of the allergic group (p = 0.0087) (**Figure 6A**). The secretion of proinflammatory cytokine IL-6 by mDCs was not increased after EcO83 stimulation. The significantly increased concentration of IL-6 in cell culture supernatants of LPS-stimulated mDCs of newborns of healthy (p = 0.0121) and allergic (p = 0.0159) mothers was not surprising. There was no difference between mDCs of newborns of healthy and allergic mothers (**Figure 6B**). The capacity of EcO83 to promote the secretion of TNF was documented for mDCs of newborns of both healthy (p = 0.0011) and allergic (p = 0.0079) mothers without any difference between healthy and allergic group. LPS stimulation significantly increased TNF secretion by mDCs of newborns of healthy (p = 0.0002) and allergic (p = 0.0079) mothers (**Figure 6C**).

EcO83 stimulation has a strong impact on the production of IL-10 by moDCs of newborns of healthy mothers (p = 0.0022). The secretion of IL-10 by moDCs of neonates of AMs was increased, but this was not statistically significant. LPS stimulation increased IL-10 release by moDCs of newborns of healthy (p = 0.0043) and allergic (p = 0.0159) mothers (**Figure 6D**). EcO83 increased the secretion of IL-6 by moDCs of newborns of healthy (p = 0.0095) and allergic (p = 0.0022) mothers. LPS stimulation increased IL-6 production only by moDCs of newborns of AMs (p = 0.0095) (**Figure 6E**). EcO83 stimulation was able to promote TNF secretion in moDCs of neonates of both healthy (p = 0.0043) and allergic (p = 0.0159) mothers. LPS stimulation promoted TNF secretion only by moDCs of newborns of healthy (p = 0.0043) mothers to a similar extent as EcO83 (**Figure 6F**).

Incubation of pDCs of newborns of healthy mothers with EcO83 and LPS stimulated the secretion of IL-10 only in the case of LPS (p = 0.0083). Stimulation of pDCs of newborns of allergic mothers with EcO83 and LPS increased IL-10 production only in the case of EcO83 (p = 0.0049). No statistically significant difference in IL-10 secretion between pDCs of newborns of healthy and allergic mothers was observed (**Figure 6G**). Neither EcO83 nor LPS stimulation has an impact on IL-6 secretion by pDCs of newborns of healthy and allergic mothers (**Figure 6H**). EcO83 stimulation promoted TNF secretion in pDCs of newborns of both healthy (p = 0.0043) and allergic (p = 0.0095) mothers. LPS stimulation led to increased TNF secretion only by pDCs of newborns of healthy mothers (p = 0.0022) (**Figure 6I**). *In vitro* stimulation of both pDCs and mDCs either directly isolated or *in vitro* derived by EcO83 significantly increased the production of key immunoregulatory cytokine IL-10. Except for LPS-induced IL-10 secretion by mDCs, no significant difference in the cytokine production by DCs between healthy and allergic groups was observed.

### 3.7 Early postnatal supplementation with Escherichia coli O83:K24:H31 does not affect the bacterial composition of 10-year-old children

The microbiome starts to set up during birth and in the first weeks of life and stabilizes at approximately the third year of life (51). Later, only environmental factors can exert a significant influence on the composition of the gut microbiome. In early infancy, the microbiome crucially regulates the immune system function. It can determine both the development and clinical course of food allergies and tolerance to food allergens. Therefore, we analyzed the influence of early postnatal supplementation with EcO83 on later gut microbiota composition in our study cohort. Ten years after EcO83 supplementation, there was no statistically significant difference in bacterial alpha diversity among any of our study groups (**Supplementary Figures S1A–S1D**). Furthermore, we did not find any significant changes in the microbiota composition among the groups (**Supplementary Figure S3**), suggesting that early postnatal EcO83 supplementation does not impact the microbiota composition later in childhood.

## 4 Discussion

Early postnatal supplementation of neonates with EcO83 appeared to lower allergy incidence in children of AMs compared with N AM. However, elevated allergy incidence was observed in boys. This observation is in agreement with previous reports documenting that preadolescent boys suffer from various kinds of allergy more frequently compared to girls (52–55). Increased levels of immunoregulatory cytokine IL-10 were observed in the sera of 10-year-old children supplemented with EcO83 compared with N AM. Treg as a critical subpopulation with regulatory function have not been influenced by EcO83 supplementation. However, PD-1 expression was significantly altered in Treg from healthy children compared with allergic ones regardless of probiotic supplementation or maternal allergy status. The possible impact of early postnatal EcO83 supplementation on the maturational status of key antigen-presenting cells in the peripheral blood of 10-year-old children has not been proven either. Interestingly, EcO83 promoted the maturation of mDCs and moDCs and the secretion of IL-10 in all types of cord blood DCs tested. Higher levels of activation marker CD83 were observed in allergic children. Therefore, increased reactivity of DCs of allergic children together with lower functional capacity of Treg could be responsible for allergy development. The gut microbiota composition at 10 years of age has not been impacted by early postnatal EcO83 supplementation.

The critical role of IL-10 in preventing allergic diseases and limiting the clinical signs of allergic disease manifestation has been acknowledged (56–59). Moreover, elevation of IL-10 in patients after successful specific allergy immunotherapy highlights the importance of IL-10 in controlling allergic disease development (56, 60). In our report, the concentration of IL-10 was increased in the sera of children supplemented with EcO83, and the level of IL-10 was comparable with concentrations in the sera of low-risk children (children of healthy mothers). A similar effect of probiotic administration on an increment of IL-10 was published by other research groups using distinct probiotic strains (61, 62). It is important to emphasize that the effect of probiotic bacteria is highly strain dependent (32). Therefore, different probiotics are suitable to promote immune responses during cancer treatment (63, 64), and different probiotics should be used to treat diseases where the promotion of immunoregulatory responses is desirable (e.g., allergy, autoimmune diseases, etc.) (65–67). Treg are considered to be an important source of IL-10 (57, 68), and some probiotic strains have been described to induce Treg both *in vitro* (33, 69) and *in vivo* (70). In the context of allergic diseases, *Lactobacillus plantarum* CJLP133 ameliorated the clinical signs of atopic dermatitis and induced Treg (71). Using experimental mouse models, the importance of probiotic strains to induce Treg has been identified as a key factor limiting allergy. The critical role of Treg in setting appropriate immunoregulatory responses has been acknowledged, but there is a controversy regarding the role of the percentage of Treg in allergic diseases. Some papers document increased levels and/or function of Treg in healthy individuals compared with allergic patients (72–74), while other studies showed no difference between these two groups (75) or even elevated levels of Treg in allergic individuals (76, 77). Therefore, not only proportion but also functional characteristics of Treg should be considered. Importantly, the functional capacity of Treg is increased in non-allergic individuals compared with that in allergic patients (43, 78). The critical role of IL-10 released by Treg has been documented using an experimental mouse model with specific deletion of IL-10 in Treg (79). The capacity of Treg to secrete cytokines with immunoregulatory function belongs to most important markers correlating with Treg function. Previously, we have reported increased IL-10+Treg and IL-10+Tr1 in children supplemented with EcO83 compared with N AM at the age of 8 years (43). Of note, increased secretion of IL-10 by Treg was associated with successful treatment of allergy (5, 80). In accordance with our previous work, no effect of EcO83 on the proportion of Treg (CD4+CD25+CD127^low^FoxP3+) was observed. In this work, we have focused on characterization of the impact of EcO83 supplementation on other markers (CTLA-4, PD-1, GITR), indicating the functional capacity of Treg. We hypothesized that EcO83 supplementation may increase the functional markers on Treg in the peripheral blood of children supplemented with EcO83 in comparison with those in non-supplemented ones, similarly to our previous observations of increased intracellular presence of IL-10 (42, 43). Contrary to our hypothesis, no significant effect of EcO83 administration on the functional markers was ascertained. Nevertheless, children suffering from allergy had a lower presence of activation markers in comparison to non-allergic ones. Our results thus suggest that, in addition to impaired immunoregulatory cytokine secretion, activation markers are downregulated in Treg of allergic individuals as well. This observation is in an agreement with the study of Pietruczuk et al. (81) who reported lower CTLA-4 and GITR in Treg of asthmatic patients on the mRNA level. CTLA-4 on Treg can downregulate the expression of CD80/CD86 on DCs, leading to the impairment of the generation allergen-specific Th2 immune responses. The important role of GITR in Treg-mediated suppression of allergen-specific Th2 immune responses was documented using a mouse experimental model (82). The other immune checkpoint PD-1 is well studied in tumor immunology. In the context of allergic diseases, a lower cell surface presence was associated with insufficient Treg function, allowing the development of allergen-specific Th2 responses in predisposed individuals (83). Our observation of elevated levels of PD-1 on Treg of healthy children compared with allergic ones is in agreement with aberrant immunoregulation in allergic persons or patients suffering from autoimmune disorders (84, 85). Nevertheless, increased levels of PD-1 on Treg were reported in patients with atopic dermatitis sensitized to food allergens compared with healthy children (86).

DCs are responsible for the induction and polarization of immune responses including the promotion of Treg. DCs are the key cellular subset responsible for priming Th2 immune responses upon allergen encounter in predisposed individuals. On the other hand, DCs can represent an important source of IL-10 contributing to the immunoregulatory environment, thus limiting adverse responses to allergen. It is still unclear what functional capacity of DC leads to preferential allergen uptake, processing, and induction of Th2 responses. We have previously observed that CD83 is the most sensitive marker pointing to DC activation after EcO83 encounter (87). In this study, the cell surface activation marker CD83 was inspected on both mDCs and pDCs in the peripheral blood of 10-year-old children. Although we expected a lower cell surface presence of CD83 in children supplemented with EcO83 in comparison with that in non-supplemented children, this effect of EcO83 stimulation on the expression of CD83 has not been proven. The only statistical difference was observed between the groups of non-allergic and allergic children for both the percentage of CD83-positive mDCs and pDCs and MFI of CD83 on both mDCs and pDCs, with CD83 always being higher in the allergic group. The increased presence of activation markers in patients suffering from allergic diseases has previously been described (88, 89). The increased presence of activation markers on DC suggests increased reactivity of both mDCs and pDCs of allergic children. This increased reactivity and/or easier activation of DCs of allergic children together with insufficient functional capacity of Treg could be responsible for the development of inadequate immune responses to environmental antigens (allergens), leading to allergic disease origination instead of inducing tolerogenic responses. The capacity of probiotic bacteria-primed DCs to induce Treg was previously documented (32, 33), but probiotic bacteria used in this study failed to increase the number of Treg when EcO83-primed DCs were cocultured with naive CD4+ T cells (87). Interestingly, CD4+ T cells cocultured with EcO83-primed DCs expressed a higher level of IL-10 in DCs of human (87) and mouse origin (90). The capacity of EcO83 to promote IL-10 is quite consistent and was observed on the level of CBMCs as well (43).

Next, we tried to confirm the capacity of EcO83 to promote the maturation of both mDCs and pDCs *in vitro*. A higher reactivity of DCs of children at higher risk for allergy development has been previously documented (58). In this study, only moDCs derived from cord blood precursors of newborns of AMs exerted a higher reactivity in comparison with moDCs of newborns of healthy mothers. Surprisingly, mDCs isolated directly from the cord blood of newborns of healthy mothers and AMs have similar levels of CD83 after both EcO83 and LPS stimulation. These results challenge the suitability of using moDCs, since we believe that directly isolated mDCs better reflect the capacity of the neonatal immune system to respond to antigens. Similarly, we tried to compare the reactivity of pDCs isolated directly from the cord blood with pDCs derived from CD34+ progenitor cells. Due to the low yield of pDCs isolated directly from cord blood, we continued only with pDCs generated *in vitro*. Interestingly, no effect of EcO83 stimulation on cell surface presence of CD83 was observed. Moreover, no difference in CD83 expression on pDCs between the healthy and the allergic group was detected. Possibly, pDCs derived *in vitro* change the epigenetic modification during 7 days of cultivation in the presence of growth factors and become uniform. In the literature, a difference in the number and function of pDCs was described directly in the lung tissue or bronchoalveolar lavages of healthy and allergic patients (91, 92). Of importance is the capacity of EcO83 to promote IL-10 secretion by DCs.

Interestingly, elevated concentrations of IL-4 were detected in the sera of N AM and S AM. If these elevated levels of IL-4 reflected a developmental stage, we believe that the group of N HM should also have been affected and increased levels of IL-4 should have been detected. This difference cannot be simply explained by inclusion of other participants then in another time intervals either, as the change in the number of participants was not remarkable over the last 3 years. It is well known that IgA production in children only reaches the adult IgA levels during later childhood (at the age of 10–15 years), and IL-4 is the typical cytokine promoting Th2 immune responses contributing to antibody production. Then again, N HM were not affected, and one would expect that these children (children at lower risk for allergy development) would be the first to increase their IgA production. In our study, EcO83 supplementation promoted levels of IgA in both sera and stool samples, suggesting that EcO83 contributes to the maturation of both systemic and mucosal immunity. The capacity of EcO83 to induce IgA secretion could contribute to the protection against infection reported previously (41). When comparing concentrations of IL-4 between healthy/non-allergic and allergic children, significantly increased levels of IL-4 were found in the allergic group (44). Because an increased number of allergic children is present in both groups of supplemented and non-supplemented children suffering from allergy compared with N HM, one can assume that the increased presence of IL-4 can simply reflect a higher number of children suffering from allergy in groups of N AM and S AM. We have reported previously that *E. coli* O83:K24:H31 has no impact on IL-4 neither *in vitro* nor *in vivo* (44, 88).

We have tried to elucidate the modes of action of early postnatal EcO83 application on the immune system in several of our previous reports. Unfortunately, we were evaluating only a limited selection of immunological compounds playing a role in allergy development (41–44). In the current report, only functional characteristics of Treg and DCs as well as cytokine concentrations were evaluated, but we acknowledge that multiple factors are playing a role in the onset of allergy. In our study, we are also limited by the source of biological material to peripheral blood, which does not always reflect the changes occurring in the gut where *Escherichia coli* O83:K24:H31 interacts with the host immune system. Notably, the understanding of the impact of *Escherichia coli* O83:K24:H31 on the mucosal immune system would be of interest. Therefore, further studies focusing on characterization of modes of action of EcO83 are needed. In particular, the impact of early postnatal EcO83 supplementation on cellular immune responses should be clarified in more detail during EcO83 supplementation when the neonatal immune system is encountering microbes colonizing the neonatal gut. Due to the low number of microbes present in the neonatal gut, EcO83 administration could have a profound effect on the development of immune responses and setting the tolerance to environmental antigens and compounds of the microbiota. With the growing knowledge on the impact of the microbiota on the immune system and development of novel methodological approaches (sequencing and microbiota analyses), we tried to compare the differences in the microbiota composition among our study groups, but we have not found any differences. The huge changes in the development of both the microbiota and the immune system are occurring in early postnatal life, but we were not able to demonstrate that the early postnatal probiotic intervention has an impact on the microbiota composition at the age of 10 years. Nevertheless, it has been demonstrated that probiotic supplementation has a limited capacity to modulate long-term microbiota composition possibly due to the colonization resistance (93). Therefore, it will be desirable to initiate an additional study on a larger cohort of participants where the impact of early postnatal EcO83 administration on the developing microbiota and neonatal immune system would be evaluated with a special focus on immune responses and mediators playing a role in the development of allergic diseases. In addition, mothers enrolled in this study have many various kinds of allergy, making it difficult to draw a strong conclusion. Perinatal antibiotic administration could represent another confounding factor. Therefore, the next trial should be designed as a double-blind placebo-controlled study, taking into account a significant drop of participants enrolled at the beginning of the trial, as well as possible perinatal antibiotic administration.

## 5 Conclusions

Early postnatal supplementation of newborns with a probiotic strain of EcO83 was able to decrease allergy incidence in the group of children of AMs in comparison with N AM. It seems that the beneficial effect of EcO83 is mediated *via* induction of the immunoregulatory cytokine IL-10. EcO83 is able to promote the maturation of mDCs directly isolated from cord blood and moDCs derived from cord blood precursor cells without impact on the maturational status of pDCs. EcO83 promoted IL-10 secretion *in vitro* by all types of DCs tested, possibly contributing to setting appropriate immunoregulatory responses in children. The diversity of the microbiota at the age of 10 years has not been affected by early postnatal supplementation with EcO83. Despite this finding, it is too early to conclusively state that the microbiota is not involved solely because it is not changed at 10 years of age among the groups of N HM and N AM and S AM. Further studies on a larger cohort will be needed to confirm our observations.

## Data availability statement

The datasets presented in this study can be found in online repositories. The name of the repository and accession number can be found below: NCBI Sequence Read Archive; PRJNA886861.

## Ethics statement

The studies involving human participants were reviewed and approved by Ethics Committee of the Institute for the Care of Mother and Child. Written informed consent to participate in this study was provided by the participants’ legal guardian/next of kin.

## Author contributions

Conceptualization, JH, RL-Ž, and LP; methodology, JH, LS, VČ, RR, TT, ZJZ, ON, PP, and ZM; formal analysis, ZV, JH, LS, VC, and KB; investigation, JH, LS, VC, TT, RR, ZJZ, ON, PP, ZM, KB, IK, and RL-Ž; resources, JH and LK; writing—original draft preparation, JH; writing—review and editing, JH, ZV, RR, ZJZ, LP, LS, LK, and VC; supervision, JH, LP, LK, and RL-Ž; project administration, JH, LK, KB, IK, and RL-Ž; funding acquisition, JH, ZJZ, ZV, and LK. All authors contributed to the article and approved the submitted version.
