# Supplementary material for: Effect of early postnatal supplementation of newborns with probiotic strain E. coli O83:K24:H31 on allergy incidence, dendritic cells, and microbiota
Source: Front Immunol. 2023 Jan 9;13:1038328. doi: 10.3389/fimmu.2022.1038328 (PMC9872645; doi:10.3389/fimmu.2022.1038328)
Supplement: Supplementary file 1 [file DataSheet_1.doc]

Supplementary Material

# Supplementary Figures and Tables

## Supplementary Figures


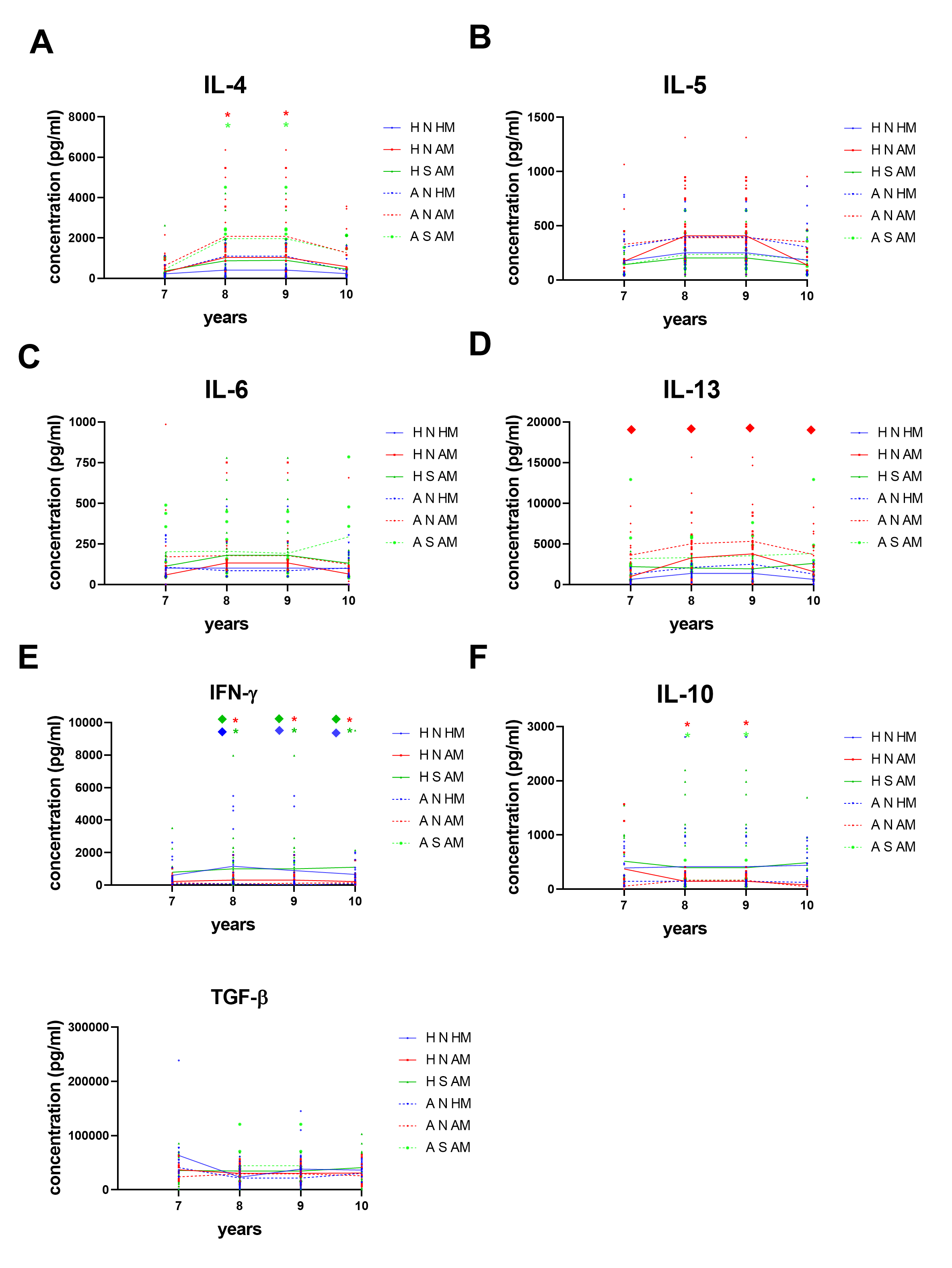


**Supplementary Figure S1. Cytokine concentration in sera.**

Cytokines were determined by ELISA. Typical Th2 cytokines were determined: IL-4 (Figure S1A), IL-5 (Figure S1B), IL-6 (Figure S1C) and IL-13 (Figure S1D). Concentration of typical Th1 cytokine (IFN-γ) is shown in Fig. S1E. Concentration of immunoregulatory cytokines is presented in Fig. S1F for IL-10 and Fig. S1G for TGF-β.

H N HM – healthy non-supplemented children of healthy mothers

H N AM – healthy non-supplemented children of allergic mothers

H S AM – healthy *E. coli* O83:K24:H31 supplemented children of allergic mothers

A N HM – allergic non-supplemented children of healthy mothers

A N AM – allergic non-supplemented children of allergic mothers

A S AM – allergic *E. coli* O83:K24:H31 supplemented children of allergic mothers


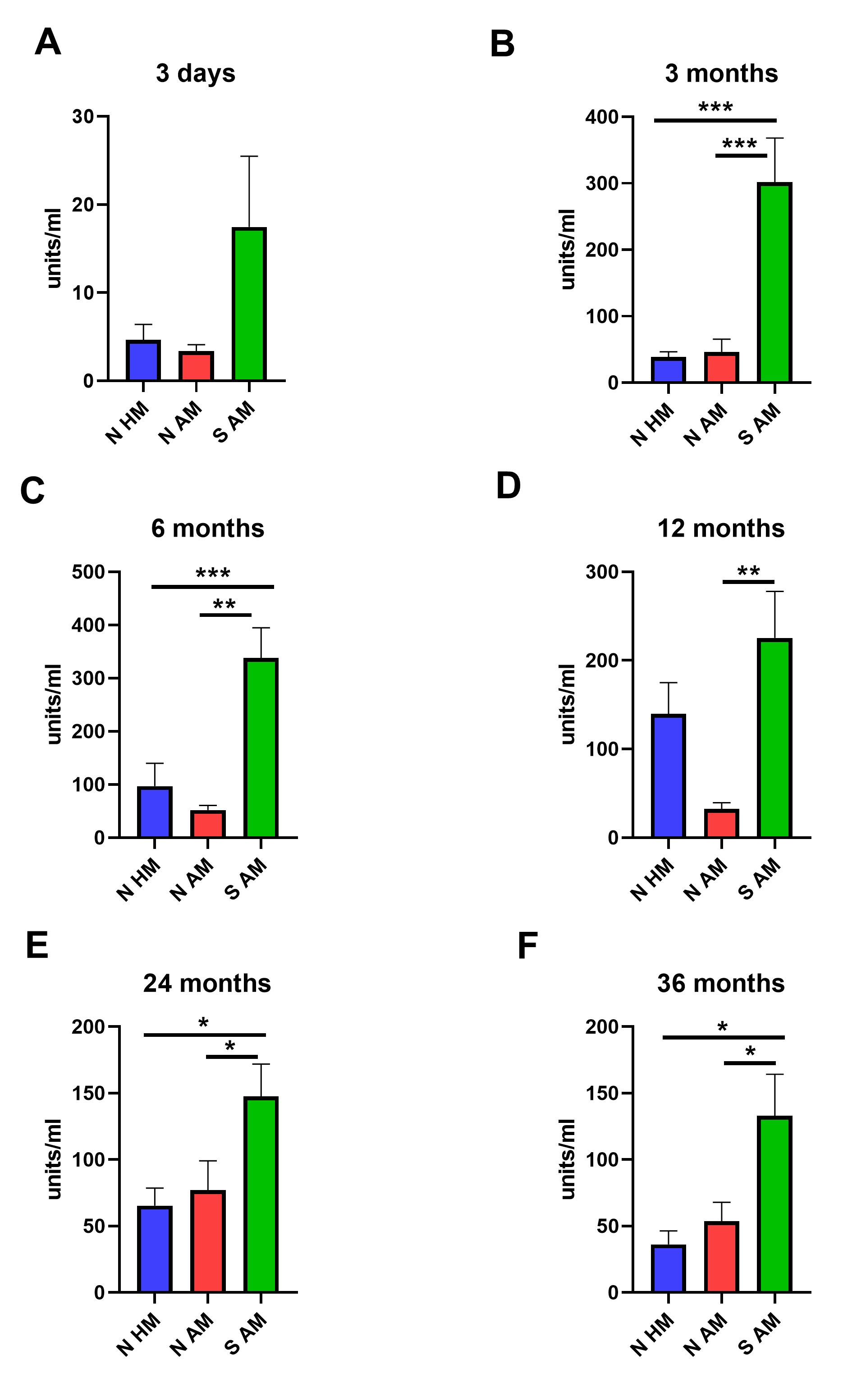


**Supplementary Figure S2. Concentration of IgA in sera.**

Concentration of IgA specific against *Escherichia coli* was detected in sera at different time points beginning on day 3 (Fig. S2A), 3 months (Fig. S2B), 6 months (Fig. S2C), 12 months (Fig. S2D), 24 months (Fig. S2E) and 36 months (Fig. S2F) by ELISA.

N HM –non-supplemented children of healthy mothers

N AM –non-supplemented children of allergic mothers

S AM –*E. coli* O83:K24:H31 supplemented children of allergic mothers


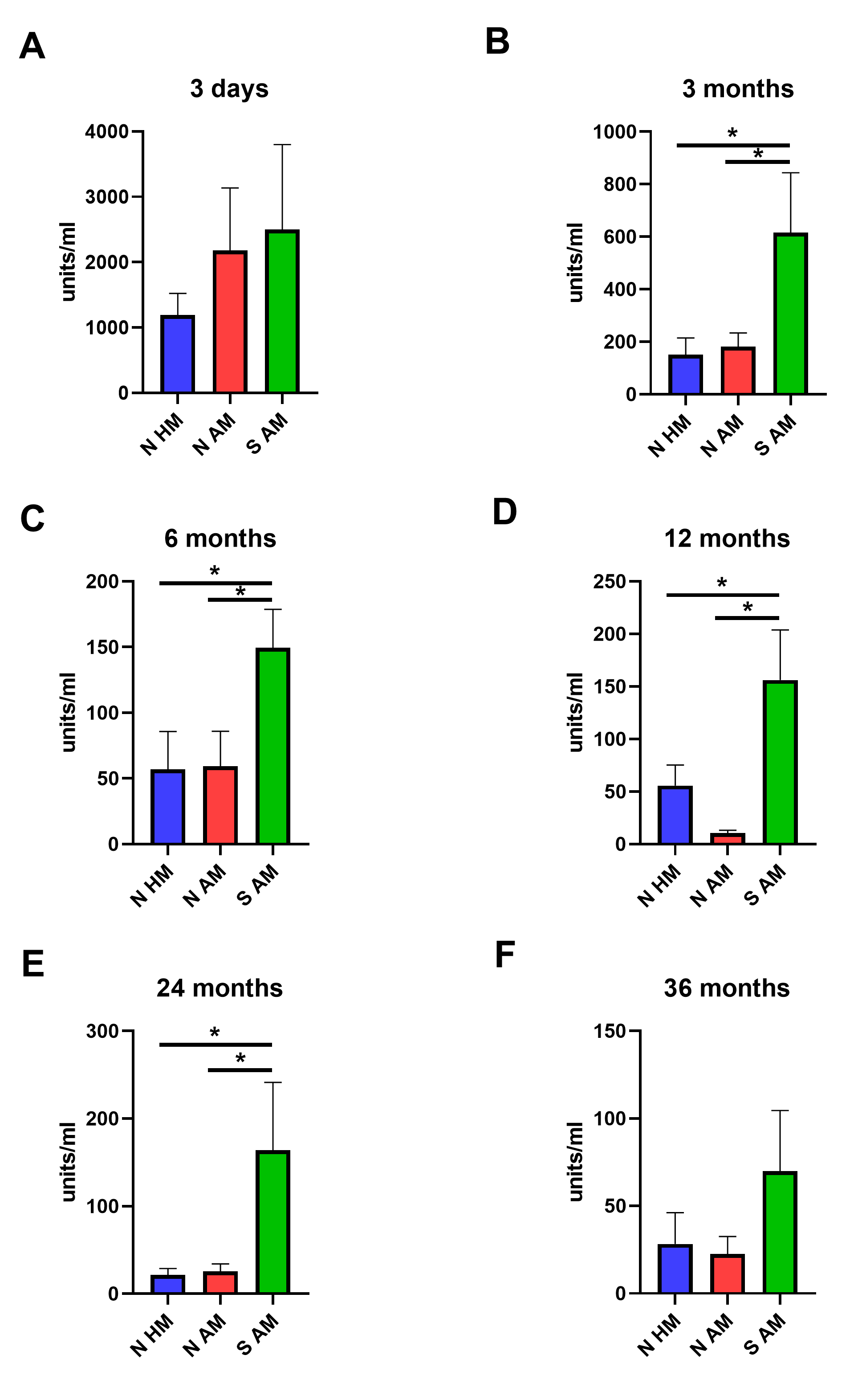


**Supplementary Figure S3. Concentration of IgA in stool.**

Concentration of IgA specific against *Escherichia coli* was detected in stool samples at different time points beginning on day 3 (Fig. S3A), 3 months (Fig. S3B), 6 months (Fig. S3C), 12 months (Fig. S3D), 24 months (Fig. S3E) and 36 months (Fig. S3F) by ELISA.

N HM –non-supplemented children of healthy mothers

N AM –non-supplemented children of allergic mothers

S AM –*E. coli* O83:K24:H31 supplemented children of allergic mothers


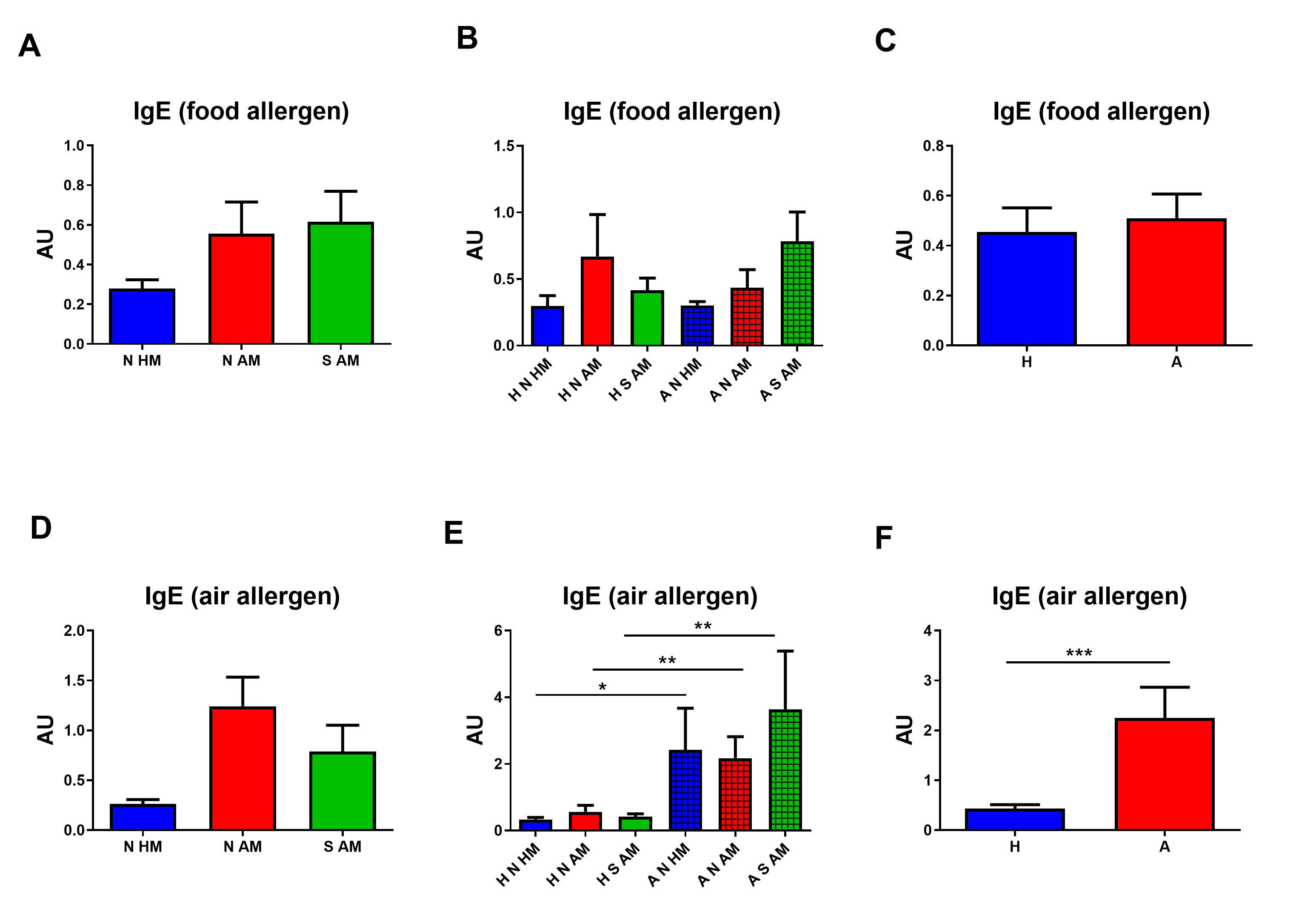


**Supplementary Figure S4. Detection IgE specific against food and respiratory allergens.**

Concentration of IgE specific against the mixture of food allergens was detected in the sera of ten-year-old children by ELISA. Concentration of IgE against food allergens was compared among the three basic groups, Fig.S4A, among the 6 subgroups, Fig.S4B and between healthy and allergic children, Fig. S4C. Concentration of IgE against respiratory allergens was compared among the three basic groups, Fig.S4D, among the 6 subgroups, Fig.S4E and between healthy and allergic children, Fig. S4F

N HM –non-supplemented children of healthy mothers

N AM –non-supplemented children of allergic mothers

S AM –*E. coli* O83:K24:H31 supplemented children of allergic mothers

H N HM – healthy non-supplemented children of healthy mothers

H N AM – healthy non-supplemented children of allergic mothers

H S AM – healthy *E. coli* O83:K24:H31 supplemented children of allergic mothers

A N HM – allergic non-supplemented children of healthy mothers

A N AM – allergic non-supplemented children of allergic mothers

A S AM – allergic *E. coli* O83:K24:H31 supplemented children of allergic mothers

A – children suffering from allergy

H – children without allergy

.

**
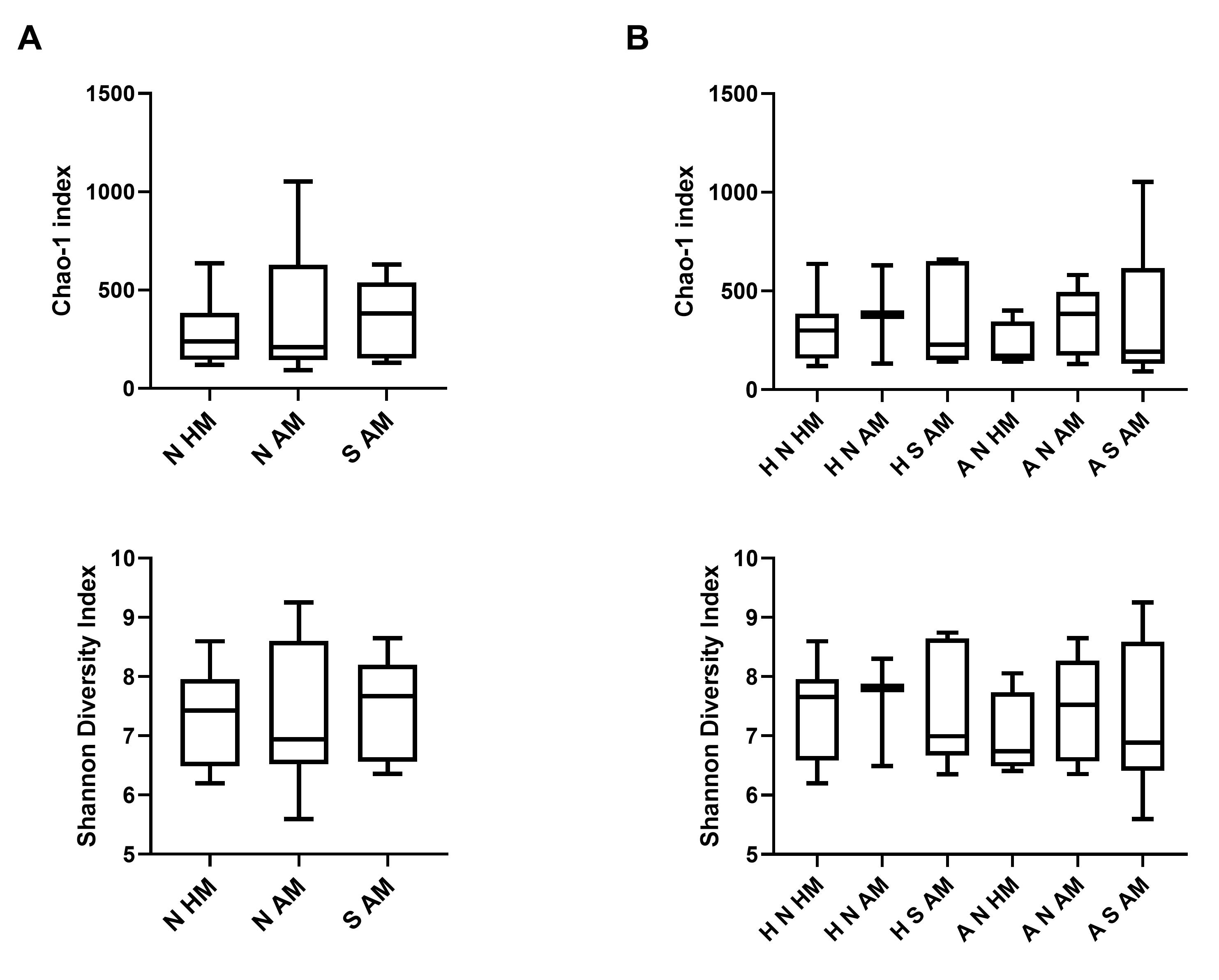
**

**Supplementary Figure S5. Difference in alpha diversity between the study groups in ten-years-old children.** Alpha diversity represented by Chao-1 index and Shannon diversity index in (A) non-supplemented children of healthy mothers (N HM), non-supplemented children of allergic mothers (N AM), and *E. coli* O83:K24:H31-supplemented children of allergic mothers (S AM) or in (B) the group of healthy non-supplemented children of healthy mothers ( H N HM), healthy non-supplemented children of allergic mothers (H N AM), healthy *E. coli* O83:K24:H31-supplemented children of allergic mothers (H S AM), allergic non-supplemented children of healthy mothers (A N HM) ), allergic non-supplemented children of allergic mothers (A N AM), allergic *E. coli* O83:K24:H31-supplemented children of allergic mothers (A S AM).

**
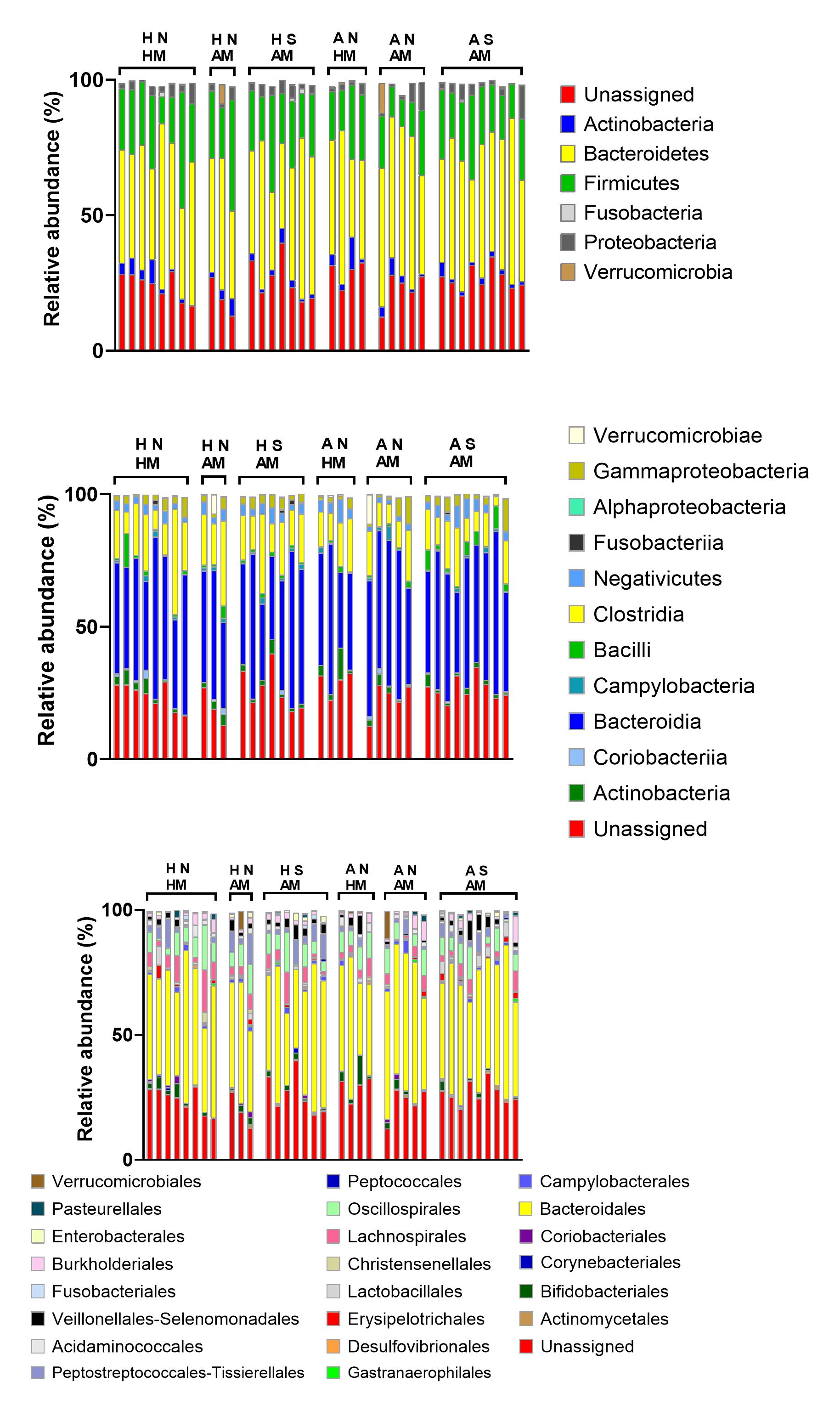
**

**Supplementary Figure 6. Stack bar charts of phylum, class, and order levels of bacterial composition** **in ten-years-old children.** The bacterial composition is shown in the group of healthy non-supplemented children of healthy mothers (H N HM), healthy non-supplemented children of allergic mothers (H N AM), healthy *E. coli* O83:K24:H31-supplemented children of allergic mothers (H S AM), allergic non-supplemented children of healthy mothers (A N HM), allergic non-supplemented children of allergic mothers (A N AM), allergic *E. coli* O83:K24:H31-supplemented children of allergic mothers (A S AM).

## Supplementary Tables

**Table S1**

List of specific allergy or combination of allergic diseases of individual children suffering from allergy confirmed by allergist.

| group | allergy outcomes (allergens) |
| --- | --- |
| A N NM | allergic rhinoconjunctivitis, bronchitis (mites, dust, grass pollen), eczema |
| A N NM | allergic rhinoconjunctivitis (rodent, grass and tree pollen) |
| A N NM | allergic rhinoconjunctivitis (dust, feathers) |
| A N NM | Allergic rhinocunjunctivitis (spring tree pollen), eczema |
| A N AM | atopic eczema, bronchitis, allergic rhinoconjunctivitis (grass pollen, dog dander, cacao, chocolate, tomatoes) |
| A N AM | atopic eczema, allergic rhinoconjunctivitis (chocolate, diary products, tomatoes, nuts, nectarines, apples) |
| A N AM | eczema, allergic rhinoconjunctivitis (grass pollen, tree pollen: birch, alder, nut tree) |
| A N AM | coeliac disease, allergic rhinoconjunctivitis (grass pollen) |
| A N AM | bronchitis (mites), eczema |
| A N AM | atopic eczema (chocolate, tomato) |
| A N AM | atopic eczema, coeliac disease, allergic rhinoconjunctivitis (mites, grass pollen, spring tree pollen) |
| A N AM | allergic rhinoconjunctivitis (mites, grass and tree pollen, animal dander) |
| A N AM | allergic rhinoconjunctivitis (grass pollen) |
| A N AM | eczema, allergic rhinoconjunctivitis (summer grass pollen) |
| A N AM | allergic rhinoconjunctivitis (cat dander, pollen) |
| A N AM | allergic rhinoconjunctivitis (birch pollen) |
| A N AM | allergic rhinoconjunctivitis (house dust mite, cat dander) |
| A S AM | atopic eczema, allergic rhinoconjunctivitis (mites, pollen, grass) |
| A S AM | allergic rhinoconjunctivitis (grass pollen, nuts, strawberries, citrus, tomatoes) |
| A S AM | allergic rhinoconjunctivitis (grass pollen) |
| A S AM | atopic eczema, allergic rhinoconjunctivitis (grass pollen) |
| A S AM | atopic eczema, allergic rhinoconjunctivitis (birch pollen, mould, peanut, egg white, cat dander) |
| A S AM | atopic eczema, allergic rhinoconjunctivitis, asthma (pollen, mould, dander, poppy seed, nuts) |
| A S AM | allergic rhinoconjunctivitis (pollen) |
| A S AM | atopic eczema, allergic rhinoconjunctivitis (pollen, dust, celery, apples) |
| A S AM | allergic rhinoconjunctivitis (spring tree pollen), atopic eczema |

A N HM – allergic non-supplemented children of healthy mothers

A N AM – allergic non-supplemented children of allergic mothers

A S AM – allergic *E. coli* O83:K24:H31 supplemented children of allergic mothers

**Table S2 Children divided into groups based on maternal** allergy status, probiotic supplementation and their allergy status at the age of 10 years.

| 3 basic groups (based on maternal allergy status and EcO83 supplementation) |  |
| --- | --- |
| N HM (n ₌ 23) | Non-supplemented children of healthy mothers |
| N AM (n ₌ 38) | Non-supplemented children of allergic mothers |
| S AM (n ₌ 45) | EcO83 supplemented children of allergic mothers |
| 6 subgroups (3 basic groups divided according to allergy status of children at the age of 10 years) |  |
| H N HM (n ₌ 19) | Healthy non-supplemented children of healthy mothers |
| A N HM (n ₌ 4; 17.4%) | Allergic non-supplemented children of healthy mothers |
| H N AM (n ₌ 25) | Healthy non-supplemented children of allergic mothers |
| A N AM (n ₌ 13; 34.2%) | Allergic non-supplemented children of allergic mothers |
| H S AM (n ₌ 36) | Healthy EcO83 supplemented children of allergic mothers |
| A S AM (n ₌ 9; 20%) | Allergic EcO83 supplemented children of allergic mothers |
| Two groups of children based only on children allergy status at the age of 10 years |  |
| A (n ₌ 26) | Allergic children |
| H (n ₌ 80) | Healthy children |

n – number of children, EcO83 – *Escherichia coli* O83:K24:H31

**Table S3**

List of specific allergy or combination of allergic diseases of individual allergic mothers divided into groups – group of non-supplemented children of allergic mothers (N AM) and *E. coli* O83:K24:H31 supplemented children of allergic mothers (S AM)

| group | allergy outcomes (allergens) |
| --- | --- |
| N AM | allergic rhinoconjunctivitis, (dust, grass pollen, feather) |
| N AM | allergic rhinoconjunctivitis (pollen, dust), eczema |
| N AM | allergic rhinoconjunctivitis (dust, pollen, mites) |
| N AM | allergic rhinoconjunctivitis (dust, pollen, mites) |
| N AM | allergic rhinoconjunctivitis (pollen, dust, mites, moulds) |
| N AM | allergic rhinoconjunctivitis (polyvalent allergy), asthma |
| N AM | eczema, allergic rhinoconjunctivitis (pollen, mites, dander), food allergy |
| N AM | allergic rhinoconjunctivitis (pollen - birch, dust, mites, feather), drug allergy (penicillin) |
| N AM | allergic rhinoconjunctivitis (pollen, dust, mites, hay), asthma, eczema |
| N AM | allergic rhinoconjunctivitis (pollen – grass), eczema |
| N AM | atopic eczema, allergic rhinoconjunctivitis (pollen, metals) |
| N AM | allergic rhinoconjunctivitis (pollen, dust, feather), food allergy (cherry, apples, nuts, carrot) |
| N AM | allergic rhinoconjunctivitis (pollen, mites, insect venom), food allergy (apples) |
| N AM | allergic rhinoconjunctivitis (pollen, dust) |
| N AM | allergic rhinoconjunctivitis (cat dander, pollen) |
| N AM | allergic rhinoconjunctivitis (birch pollen) |
| N AM | allergic rhinoconjunctivitis (house dust mite, cat dander) |
| N AM | atopic eczema, allergic rhinoconjunctivitis (mites, pollen, grass) |
| N AM | allergic rhinoconjunctivitis (grass pollen, nuts, strawberries, citrus, tomatoes) |
| N AM | allergic rhinoconjunctivitis (grass pollen) |
| N AM | asthma, allergic rhinoconjunctivitis (pollen, feather) |
| N AM | asthma, allergic rhinoconjunctivitis (pollen, feather), food allergy (nuts, apples, tropical fruits) |
| N AM | asthma, eczema, allergic rhinoconjunctivitis, asthma (pollen, dust, incect venom) |
| N AM | allergic rhinoconjunctivitis (pollen – grass, dust, feather), asthma |
| N AM | ezema, allergic rhinoconjunctivitis (pollen, dust, feather), asthma |
| N AM | asthma (dust, moulds), drug allergy - penicillin) |
| N AM | allergic rhinoconjunctivitis (dust, pollen – timothy grass, rye) |
| N AM | allergic rhinoconjunctivitis |
| N AM | allergic rhinocunjunctivitis (pollen, dust, mites) |
| N AM | bronchitis, allergic rhinoconjunctivitis (grass pollen, mites, dust) |
| N AM | asthma, allergic rhinoconjunctivitis (pollen, dust, exotic birds) |
| N AM | asthma, allergic rhinoconjunctivitis (pollen, dust), food allergy (nuts) |
| N AM | allergic rhinoconjunctivitis (grass pollen, dust), asthma |
| N AM | asthma (pollen, dust,feather) |
| N AM | atopic eczema (chocolate, tomato) |
| N AM | allergic rhinoconjunctivitis (mites, grass pollen, mites, dust) |
| N AM | allergic rhinoconjunctivitis (mites, grass and tree pollen, animal dander) |
| N AM | allergic rhinoconjunctivitis (grass pollen) |
| N AM | eczema, allergic rhinoconjunctivitis (summer grass pollen) |
| N AM | allergic rhinoconjunctivitis (pollen. dust) |
| N AM | allergic rhinoconjunctivitis (birch pollen, mites), food allergy (apples) |
| N AM | asthma, allergic rhinoconjunctivitis (house dust mite, cat dander, pollen) |
| S AM | atopic eczema, allergic rhinoconjunctivitis (mites, pollen, dust, feather, horses, insect venom), drug allergy - penicillin |
| S AM | asthma, allergic rhinoconjunctivitis (pollen, dust, feather), food allergy (chocolate) |
| S AM | asthma, allergic rhinoconjunctivitis (pollen, dust, feather), food allergy (chocolate) |
| S AM | allergic rhinoconjunctivitis (pollen, dust, feather) |
| S AM | allergic rhinoconjunctivitis (pollen, mites, dust, feather, hay), eczema, asthma |
| S AM | eczema, allergic rhinoconjunctivitis (pollen), food allergy (nuts, apples, kiwi) |
| S AM | eczema, allergic rhinoconjunctivitis (dust, pollen), food allergy (cow milk) |
| S AM | allergic rhinoconjunctivitis, asthma |
| S AM | allergic rhinoconjunctivitis (pollen, dust, mites, feather, hay), eczema, asthma, food allergy (fishes) |
| S AM | allergic rhinoconjunctivitis (cat, dander, dust, moulds, grass pollen), atopic dermatitis |
| S AM | atopic eczema, allergic rhinoconjunctivitis (birch pollen, mould, peanut, egg white, cat dander) |
| S AM | asthma, allergic rhinoconjunctivitis (house dust mite, pollen) |
| S AM | asthma, allergic rhinoconjunctivitis (house dust mite, pollen) |
| S AM | allergic rhinoconjunctivitis (pollen – birch, dust), eczema, asthma |
| S AM | allergic rhinoconjunctivitis (dust, mites, pollen) |
| S AM | atopic eczema, allergic rhinoconjunctivitis (pollen, dust, feather), food allergy (milk, tomato, potato, tropical fruits) |
| S AM | atopic eczema, allergic rhinoconjunctivitis (pollen, dust, feather), food allergy (milk, tomato, potato, tropical fruits) |
| S AM | asthma, allergic rhinoconjunctivitis (house dust mite, pollen), drug allergy – penicillin |
| S AM | atopic eczema, asthma, allergic rhinoconjunctivitis (pollen - birch) |
| S AM | allergic rhinoconjunctivitis (pollen, dust, feather), asthma |
| S AM | asthma, allergic rhinoconjunctivitis (house dust mite,feather, pollen), food allergy |
| S AM | allergic rhinoconjunctivitis (mites, pollen, feather, moulds), asthma |
| S AM | allergic rhinoconjunctivitis (mites, pollen, feather, moulds), asthma |
| S AM | allergic rhinoconjunctivitis (mites, pollen, feather, moulds), asthma |
| S AM | asthma, allergic rhinoconjunctivitis (pollen, dust, mites) |
| S AM | allergic rhinoconjunctivitis (pollen, mites, moulds), drug allergy |
| S AM | asthma, allergic rhinoconjunctivitis (house dust mite, feather, moulds, pollen) |
| S AM | atopic eczema, allergic rhinoconjunctivitis (mites, pollen, grass) |
| S AM | atopic eczema, allergic rhinoconjunctivitis (mites, pollen, grass) |
| S AM | asthma, atopic eczema, allergic rhinoconjunctivitis (mites, pollen, grass) |
| S AM | asthma, allergic rhinoconjunctivitis (mites, dust, feather, pollen: birch, grass) |
| S AM | allergic rhinoconjunctivitis (pollen, dust, moulds, feather), eczema |
| S AM | allergic rhinoconjunctivitis (pollen, feather, dust, mites), asthma, eczema |
| S AM | asthma (pollen, feather, mites), food allergy - nuts |
| S AM | allergic rhinoconjunctivitis (mites, pollen, dust, insect venom, hay), food allergy (nuts), drug allergy - penicillin |
| S AM | asthma, allergic rhinoconjunctivitis (house dust mite, feather, pollen, insect venom, hay) |
| S AM | atopic eczema, allergic rhinoconjunctivitis (mites, pollen, grass) |
| S AM | allergic rhinoconjunctivitis (pollen, dust, mites) |
| S AM | asthma, allergic rhinoconjunctivitis (house dust mite, cat dander, pollen) |
| S AM | allergic rhinoconjunctivitis (mite, pollen) |
| S AM | allergic rhinoconjunctivitis (pollen, feather, fur) |
| S AM | allergic rhinoconjunctivitis (pollen: timothy grass, ray, wormwood,), asthma |
| S AM | atopic eczema, allergic rhinoconjunctivitis (grass pollen timothy grass, wormwood) |
| S AM | allergic rhinoconjunctivitis (birch pollen, mites), food allergy (apples) |
| S AM | allergic rhinoconjunctivitis (pollen), food allergy - sugar |
| S AM | allergic rhinoconjunctivitis (mites, pollen, grass) |
| S AM | allergic rhinoconjunctivitis (pollen, moulds), food allergy (nuts, strawberries, tomatoes), eczema |
| S AM | asthma, allergic rhinoconjunctivitis (house dust mites, pollen) |
| S AM | atopic eczema, allergic rhinoconjunctivitis (mites, pollen, grass, moulds) |
| S AM | allergic rhinoconjunctivitis (pollen, dust) |
| S AM | atopic eczema, allergic rhinoconjunctivitis (grass and spring tree pollen) |
| S AM | allergic rhinoconjunctivitis (mite, pollen, dust), food allergy (apples) |

N AM – allergic mothers of non-supplemented children

S AM – allergic allergic mothers of *E. coli* O83:K24:H31 supplemented children

Table S4 Perinatal characteristics of children included in the study.

| group | Length of pregnancy | Birth weight (kg) | Birth length (cm) | Age of birth (years) |
| --- | --- | --- | --- | --- |
| N HM | 39 w 5/7 ± 7.8 | 3.533±0.344 | 50.9±1.6 | 30.4±2.8 |
| N AM | 39 w 5/7 ± 7.7 | 3.447±0.335 | 50.0±1.4 | 30.2±2.7 |
| S AM | 40 w 0/7 ± 6.1 | 3.366±0.338 | 49.8±1.3 | 30.3±3.2 |

Values represent mean±standard error mean.

N HM - Non-supplemented children of healthy mothers

N AM- Non-supplemented children of allergic mothers

S AM – *E. coli* O83:K24:H31 supplemented children of allergic mothers

**Table S5 Children divided into groups based on maternal allergy status, probiotic supplementation and their allergy status at the age of 10 years. All children with known allergic status were inclu**ded.

| 3 basic groups (based on maternal allergy status and EcO83 supplementation) |  |
| --- | --- |
| N HM (n ₌ 32) | Non-supplemented children of healthy mothers |
| N AM (n ₌ 42) | Non-supplemented children of allergic mothers |
| S AM (n ₌ 52) | EcO83 supplemented children of allergic mothers |
| 6 subgroups (3 basic groups divided according to allergy status of children at the age of 10 years) |  |
| H N HM (n ₌ 26) | Healthy non-supplemented children of healthy mothers |
| A N HM (n ₌ 6; 18.8%) | Allergic non-supplemented children of healthy mothers |
| H N AM (n ₌ 26) | Healthy non-supplemented children of allergic mothers |
| A N AM (n ₌ 16; 38.1%) | Allergic non-supplemented children of allergic mothers |
| H S AM (n ₌ 39) | Healthy EcO83 supplemented children of allergic mothers |
| A S AM (n ₌ 13; 25%) | Allergic EcO83 supplemented children of allergic mothers |

n – number of children, EcO83 – *Escherichia coli* O83:K24:H31
